# Combined treatment with *Acorus tatarinowii* Schott and *Panax notoginseng* saponins ameliorates brain–gut axis dysfunction in MCAO/R rats with suppression of TLR4/MyD88/NF-κB signaling and associated gut microbiota changes

**DOI:** 10.3389/fphar.2026.1683558

**Published:** 2026-06-29

**Authors:** Lin-Yao Hao, Zhi-Peng Huang, Hui-Min Zhang, Yu-Ting Pu, Ling-Xue Wang, Hong-Mei Tang, De-Chou Zhang, Xue Bai, Shuang-Yang Li

**Affiliations:** 1 Department of Neurology, National Traditional Chinese Medicine Clinical Research Base, The Affiliated Traditional Chinese Medicine Hospital of Southwest Medical University, Luzhou, Sichuan, China; 2 Institute of Integrated Chinese and Western Medicine, Southwest Medical University, Luzhou, Sichuan, China

**Keywords:** *Acorus tatarinowii* Schott, brain-gut axis, combination therapy, gut microbiota, ischemic stroke, *Panax notoginseng* saponins, TLR4/MyD88/NF-κB pathway

## Abstract

**Background and purpose:**

Cerebral ischemia-reperfusion injury disrupts the brain-gut axis, which involves the activation of the toll-like receptor 4 (TLR4)/myeloid differentiation factor 88 (MyD88)/nuclear factor-kappa B (NF-κB) pathway and gut microbiota dysregulation. *Acorus tatarinowii* Schott (AT) and *Panax notoginseng* saponins are traditional Chinese medicines with neuroprotective potential, yet the mechanism underlying their combined action remains elusive.

**Materials and methods:**

144 Sprague-Dawley rats were divided into groups including: sham, model (transient middle cerebral artery occlusion/reperfusion, MCAO/R), and drug-treated groups receiving *A. tatarinowii* Schott (AT), *P. notoginseng* saponins (PNS), or their combination (PAT). To evaluate efficacy under heightened inflammatory conditions, parallel groups were pre-treated with lipopolysaccharide (LPS, a TLR4 pathway agonist) before MCAO/R and subsequent drug administration (e.g., LPS + MCAO/R, LPS + AT, LPS + PNS, LPS + PAT, etc.). Neurological deficits (Longa score), cerebral infarct volume (TTC staining), intestinal motility, and histopathology (Hematoxylin-eosin staining, transmission electron microscopy) were analyzed. Additionally, levels of tight junction proteins (ZO-1, claudin-5, occludin), TLR4 pathway components, and inflammatory cytokines (TNF-α, IL-6) were assessed using Western blot and/or immunohistochemistry. Gut microbiota composition was evaluated via 16S rDNA sequencing.

**Results:**

Compared with either monotherapy, the combined AT–PNS treatment produced greater improvements in neurological function, reduced cerebral infarct volume, preserved blood-brain and intestinal barrier integrity, and attenuated TLR4/MyD88/NF-κB pathway activation. The combination was also associated with partial normalization of cecal microbiota composition, including an increased Firmicutes/Bacteroidota ratio and reduced Proteobacteria abundance. These effects were attenuated but remained detectable under LPS-induced inflammatory challenge.

**Conclusion:**

Combined AT–PNS treatment ameliorated brain-gut axis dysfunction after cerebral ischemia-reperfusion injury and showed greater efficacy than either monotherapy in this fixed-dose experimental design. These effects were accompanied by suppression of TLR4/MyD88/NF-κB signaling, improved barrier integrity, and associated changes in gut microbiota composition. Because the present study did not include dose-response matrices, a formal dose-response interaction profile and a causal microbiota-TLR4 mechanism remain to be established in future studies.

## 1 Introduction

Ischemic stroke (IS) remains a leading cause of global morbidity and mortality and is characterized by complex pathophysiological cascades, including oxidative stress, neuroinflammation, and blood-brain barrier (BBB) disruption. These processes exacerbate neuronal damage and contribute to poor clinical outcomes (Campbell et al., 2019; Sloane and Camargo, 2019). Beyond cerebral injury, emerging evidence highlights the critical role of the brain-gut axis (BGA) in stroke pathophysiology. Clinically, over 50% of stroke patients experience significant gastrointestinal complications, including delayed gastric emptying, intestinal motility disorders, and gut barrier dysfunction, which are associated with increased risk of infection and poor prognosis (Zhai, 2022). The duodenum, as the primary site for nutrient absorption and a region with dense immunological and neural networks, is particularly vulnerable to ischemic and inflammatory insults propagated from the brain (Brea et al., 2021). Its rapid response to systemic stress makes it an ideal location to investigate early-stage gut injury and barrier failure in the context of cerebral ischemia. Therefore, this study specifically focused on the duodenum to assess the impact of Cerebral ischemia-reperfusion injury (CIRI) on the gut and the protective effects of our therapeutic intervention.

Traditional Chinese Medicine (TCM) emphasizes holistic regulation and multi-target intervention, offering potential advantages in managing complex diseases such as ischemic stroke. *Panax notoginseng* saponins (PNS), a well-established neuroprotective agent, faces translational limitations related to limited BBB penetration and incomplete targeting of post-stroke brain-gut axis dysfunction. Conversely, *Acorus tatarinowii* Schott (AT), a “wind medicine” in TCM, has been reported to enhance BBB tight junction protein expression and attenuate TLR4/MyD88/NF-κB pathway activation, thereby reducing neuroinflammatory responses. Additionally, it has also been associated with improved intestinal motility and changes in gut microbial composition, and facilitates the absorption of other medicinal compounds in the intestinal tract (Gilani et al., 2006; Xiong et al., 2022; Wang et al., 2010; Liu et al., 2020). Given AT’s capacity to enhance BBB integrity and mitigate a key neuroinflammatory pathway, we reasoned that its combination with PNS could theoretically overcome the latter’s limitations and produce greater therapeutic effects than either treatment alone. However, the mechanisms underlying their combined effects on brain-gut axis regulation remain unexplored.

This study integrates TCM’s holistic philosophy with modern mechanistic research to investigate the combined effects of AT and PNS in a transient middle cerebral artery occlusion/reperfusion (MCAO/R) rat model. We hypothesized that the combined effects of AT and PNS may be associated with attenuation of neuroinflammation, improvement of intestinal barrier integrity, and accompanying changes in gut microbial composition. Our findings indicate that the combination therapy produced greater effects than monotherapy in reducing infarct volume and improving neurological function, and was associated with partial normalization of gut microbial composition, although these beneficial effects were partially attenuated under LPS-induced inflammatory challenge. This work provides novel insights into TCM’s multi-organ regulatory capacity and establishes a scientific foundation for combination therapies targeting the brain-gut axis in IS management. While our prior work focused on BBB restoration, this study extends the investigation to the systemic brain-gut axis, examining combined effects on neuroinflammation, intestinal integrity, and gut microbial composition, thereby offering a more holistic understanding of its therapeutic potential.

## 2 Materials and methods

### 2.1 Materials

#### 2.1.1 Drugs and authentication

The botanical drug material consisted of the dried rhizome of *A. tatarinowii* Schott (authority: Schott) [Acoraceae; *Acori Tatarinowii* Rhizoma], commonly known as “Shi Chang Pu” in TCM. According to TCM processing standards, the crude drug was cleaned, sliced, and dried prior to extraction, consistent with the processing methods recorded in the Chinese Pharmacopoeia for this medicinal material. The collection and use of *A. tatarinowii* Schott crude drug complied with all relevant national and international regulations. The material was sourced from a licensed commercial supplier in China, ensuring that its procurement complied with the Nagoya Protocol and Convention on International Trade in Endangered Species of Wild Fauna and Flora on Access and Benefit-sharing. All necessary phytosanitary certificates were obtained for the transportation of the material.

*Acorus tatarinowii* Schott (AT) crude drug was purchased from Sichuan Traditional Chinese Medicine Drinking Tablets Co. Ltd. (Chengdu, China). The identity of the botanical drug was authenticated by Prof. Qing-Rong Pu, a Chief Chinese Pharmacist at the Affiliated Traditional Chinese Medicine Hospital of Southwest Medical University, via macroscopic and microscopic examination against voucher specimens and authoritative references. The aqueous extract was prepared by decocting the crude drug twice in distilled water at a 10:1 volume-to-weight ratio for 1 h each time, followed by filtration, concentration, and freeze-drying. The final extraction yield was 9.8%. The AT extract was characterized using orthogonal analytical approaches, including targeted HPLC-DAD quantification of α-asarone and β-asarone and untargeted Q-Orbitrap high-resolution LC-MS/MS profiling. The chromatographic conditions, representative chromatograms, marker-compound quantification, total ion chromatograms, representative database-matched compounds, and the inventory of original analytical reports are provided in Supplementary File S1 and Supplementary Data Files S1A–S1E. The resulting aqueous extract of *A. tatarinowii* Schott was a brown powder with a characteristic aromatic odor. The extract was assigned a unique batch number (AT20210915), with the date of production recorded as 15 September 2021. It was stored desiccated at −20 °C in airtight containers, and its chemical stability under these conditions was confirmed for the duration of the study.

*Panax notoginseng* saponins (PNS, Xuesaitong Injection, Freeze-dried, Kunming Pharmaceutical Group, China; State Pharmaceutical License: Z20026438, Batch No. 2109B37) were dissolved in saline (4 mg/mL) before use.

#### 2.1.2 Reagents

Lipopolysaccharide (LPS, Sigma, United States), 2,3,5-triphenyl tetrazolium chloride (TTC, Solarbio, China), and ELISA kits for IL-6 and TNF-α (Neobioscience, China) were used. Primary antibodies (TLR4 and ZO-1, Santa Cruz, United States; MyD88, NF-κB p65, mAb IgG, and β-actin, Cell Signaling Technology, United States; TNF-α, Proteintech, United States; IL-6, BOSTER, China; Occludin and claudin-5, Invirogen, United States) and secondary antibodies (BioX, United States) were also employed.

#### 2.1.3 Instruments

A Leica optical microscope (Germany), a transmission electron microscope (JEM-1400FLASH TEM, Japan), a ChemiDoc™ XRS + System gel imaging instrument (Bio-Rad, United States), and an MCAO wire bolt (Beijing XINON, China) were utilized.

### 2.2 Animal model and experimental design

#### 2.2.1 Animals

Male Sprague-Dawley rats (n = 144, weighing 250–280 g, SPF grade) were obtained from the Southwest Medical University Animal Center (Laboratory Animal Production License: SYXK(Chuan) 2023-0017; Use License: SYXK(Chuan) 2023-0065). Rats were housed under standard conditions (22 °C ± 2 °C, humidity of 45% ± 5%, 12 h light/dark cycle) with *ad libitum* access to food and water. All procedures were conducted in accordance with ARRIVE guidelines and were approved by the Laboratory Animal Ethics Committee of Southwest Medical University (No. 20220225-006).

#### 2.2.2 Middle cerebral artery occlusion/reperfusion (MCAO/R) model

The middle cerebral artery occlusion/reperfusion (MCAO/R) model was established using the intraluminal filament technique. Adult male Sprague-Dawley rats were fasted overnight prior to surgery. Anesthesia was induced with sodium pentobarbital (40 mg/kg, i.p.), and body temperature was maintained at 37.0 °C ± 0.5 °C using a heating pad throughout the procedure. Following a midline neck incision, the right common carotid artery (CCA), external carotid artery (ECA), and internal carotid artery (ICA) were carefully isolated from the adjacent vagus nerve and fascia. The CCA was temporarily occluded by a knot with a 6–0 silk suture. The ECA was then ligated and cut, leaving a stump. A silicon-coated monofilament (MCAO wire bolt, Beijing XINON, China) was introduced into the ECA stump and advanced into the ICA until mild resistance was felt, approximately 18–20 mm from the carotid bifurcation, to occlude the origin of the middle cerebral artery. Immediately upon awakening from anesthesia, successful occlusion was confirmed by the observation of neurological deficits, such as contralateral forelimb flexion when the animal was suspended by the tail. The filament remained in place for 2 h to maintain MCA occlusion. For reperfusion, the rat was briefly re-anesthetized. The filament was carefully withdrawn back into the ECA stump to restore blood flow to the MCA territory, and then the knot on the CCA was released to restore blood flow through the CCA. The ECA stump was then permanently ligated. Patency of the CCA was visually confirmed. Sham-operated rats underwent identical procedures, except for the insertion of the filament.

#### 2.2.3 Experimental groups and drug administration

144 SD rats were divided into nine experimental groups (n = 16 per group): sham, model (transient middle cerebral artery occlusion/reperfusion, MCAO/R), and drug-treated groups receiving *A. tatarinowii* Schott (AT), *P. notoginseng* saponins (PNS), or their combination (PAT). To evaluate efficacy under heightened inflammatory conditions, parallel groups were pre-treated with lipopolysaccharide (LPS, a TLR4 pathway agonist) before MCAO/R and subsequent drug administration (e.g., LPS + MCAO/R, LPS + AT, LPS + PNS, LPS + PAT, etc.). LPS was selected as a canonical TLR4 agonist to simulate endotoxin exposure and rigorously evaluate the resilience of the treatments under a controlled inflammatory stress. In the LPS-pre-treated groups, LPS (50 μg/kg) was administered via intraperitoneal (i.p.) injection 2 h before surgery.

Following surgery, the treatment regimens were as follows: the AT group received AT (1.56 g/kg/day) by gavage; the PNS group received PNS (20.8 mg/kg/day, i. p.); the PAT group received both AT (1.56 g/kg/day, gavage) and PNS (20.8 mg/kg/day, i. p.); the LM group received LPS plus saline; the LAT group received LPS plus AT; the LP group received LPS plus PNS; and the LPAT group received LPS plus PAT. The sham and MCAO/R control groups received equivalent volumes of saline. Drugs were administered once daily for 7 consecutive days starting immediately after reperfusion.

The intraperitoneal route was employed for PNS administration to ensure consistent and efficient systemic delivery in rodents, a standard approach in preclinical pharmacokinetic studies (Yang and Zhao, 2024). This allows for direct comparison with foundational research, although we acknowledge the difference from the clinical intravenous route as a translational consideration discussed later.

In addition to the 144 rats used in the main efficacy experiment, a separate pilot tolerability assessment was performed in sham-operated rats treated with AT, PNS, or AT–PNS using the same dosing regimen. These animals were used only for tolerability evaluation and were not included in the efficacy analyses. Compared to saline-treated sham animals, rats in the drug-administered groups showed no significant alterations in body weight, general behavior, or serum biochemical indicators of hepatic and renal function, including alanine aminotransferase (ALT), aspartate aminotransferase (AST), blood urea nitrogen (BUN), and creatinine (CREA). These results indicate that the treatments were well-tolerated at the administered doses. The corresponding data are presented in the Supplementary Material (Supplementary Table S1).

### 2.3 Experimental procedures

#### 2.3.1 Neurological assessment

Neurological deficits were evaluated after the final drug administration on day 7 using the Longa score (0–4 scale): 0, no deficits; 1, left forelimb flexion; 2, circling to the left when walking; 3, falling to the left; 4, loss of consciousness (Longa et al., 1989).

#### 2.3.2 Sample collection and intestinal motility assessment

Following 12-h fasting, rats were gavaged with 10 mL/kg semi-solid ink paste (containing 5% activated charcoal). After 30 min, anesthesia was induced by intraperitoneal pentobarbital (40 mg/kg). Blood was collected via abdominal aorta puncture prior to decapitation on ice. Brain tissues, duodenum, and cecal contents were rapidly excised. The total intestinal length (L) from pylorus to ileocecum and ink propulsion distance (L0) were measured to calculate propulsion rate (L0/L×100%) (Tang et al., 2021). Tissues were either snap-frozen at −80 °C or fixed in 4% paraformaldehyde.

#### 2.3.3 Determination of cerebral infarction rate

Cerebral infarction volume was assessed after 7 days of drug administration using 2,3,5-triphenyltetrazolium chloride (TTC) staining. Briefly, animals were deeply anesthetized with sodium pentobarbital (100 mg/kg, i.p.) and decapitated. The brains were rapidly removed and placed on a chilled brain matrix pre-cooled to −20 °C for 5 min to facilitate slicing. The olfactory bulbs and cerebellum were removed. The brain was then sectioned coronally into five consecutive 2-mm-thick slices (beginning from the frontal pole) using a sharp razor blade. The brain slices were immediately immersed in a commercially available pre-prepared 2% TTC solution (Solarbio, China) and incubated at 37 °C in the dark for 20 min with gentle agitation. After incubation, the slices were transferred to 4% paraformaldehyde (PFA) for fixation at 4 °C for 24 h to enhance contrast and preserve tissue integrity. The stained slices were then photographed, and image analysis was performed using ImageJ software by an investigator blinded to group allocation. The infarct area (pale, unstained region) was measured for each slice. The total infarct volume was calculated as the sum of infarct areas multiplied by slice thickness (2 mm). Infarct rate was expressed as a percentage of total brain volume calculated as (total infarct volume/total brain volume) × 100%.

#### 2.3.4 Histopathological examination

The duodenum was selected for histopathological analysis due to its high susceptibility to ischemic insult and its key role in brain-gut communication. To ensure consistency and comparability across all experimental groups, a standardized segment of the proximal duodenum (approximately 2 cm in length, located 1–3 cm distal to the pylorus) was collected for all subsequent histopathological and molecular analyses.

Paraffin-embedded brain and duodenal tissues fixed in 4% paraformaldehyde (>24 h) were sectioned at 5 μm. Hematoxylin-eosin (HE) staining was performed for histopathological evaluation under light microscopy at a magnification of ×100. Duodenal mucosal injury was evaluated using a modified histological scoring system based on previously established criteria (Koelink et al., 2018; Fu et al., 2022), with the following parameters each scored from 0 (normal) to 3 (severe) and total 0–15:

- Villus architecture: 0, normal (villus height/crypt depth ratio >3:1); 1 mild villus shortening (ratio 2–3:1); 2, moderate villus atrophy (ratio 1–2:1); 3, severe villus atrophy or loss (ratio <1:1);
- Epithelial integrity: 0, intact epithelium; 1, focal epithelial detachment; 2, multifocal epithelial loss; 3, extensive epithelial denudation;
- Goblet cell loss: 0, normal density; 1 ≤ 25% loss; 2, 25%–50% loss; 3 ≥ 50% loss;
- Inflammatory cell infiltration: 0, absent; 1, mild (scattered cells in lamina propria); 2, moderate (confluent infiltration in lamina propria); 3, severe (infiltration extending to submucosa);
- Crypt hyperplasia: 0, normal; 1, mild increase in crypt depth; 2, moderate hyperplasia; 3, severe hyperplasia.

#### 2.3.5 Ultrastructural analysis

Brain tissues from ischemic hemispheres and duodenum tissues were fixed with 3% glutaraldehyde and 1% osmium tetroxide, dehydrated through acetone gradients, and embedded in Ep812. Ultrathin sections (60–90 nm), stained with uranyl acetate-lead citrate, were examined using JEM-1400FLASH transmission electron microscopy (TEM). The integrity of the blood-brain barrier (BBB) was assessed ultrastructurally by examining the continuity of the endothelial cells, the presence of tight junctions, the state of the basement membrane (e.g., thickening or dissolution), and the degree of perivascular edema. Duodenal mucosal barrier integrity was evaluated by observing the structure of epithelial cell microvilli (e.g., orderly arrangement vs. shedding), the morphology of intercellular tight junctions (e.g., closed vs. open), and the condition of organelles such as mitochondria.

#### 2.3.6 Immunohistochemistry

The experimental procedures for immunohistochemistry were as described: Duodenal sections underwent antigen retrieval and 5% BSA blocking. Primary antibodies against ZO-1 (1:100) and NF-κB p65 were incubated overnight at 4 °C, followed by HRP-conjugated secondary antibodies (1:5,000) at 37 °C for 1 h. DAB chromogen and hematoxylin counterstaining were performed. Specifically for NF-κB p65 staining: The primary antibody used was Anti-NF-κB p65 (D14E12) Rabbit monoclonal antibody (Cell Signaling Technology, Danvers, MA, United States; Catalog # 8242). It was applied at a working concentration of 1:500 dilution for immunohistochemistry, as recommended by the manufacturer.

ImageJ-based quantification of NF-κB p65 positive rate: Positive cells were quantified at ×200 magnification using ImageJ software (version 1.53c). The analysis pipeline was executed as follows: First, the acquired digital images were calibrated and converted to 8-bit grayscale. Three non-overlapping fields per section were randomly selected. Within each field, the epithelial layer and lamina propria were manually outlined as separate Regions of Interest (ROIs). For cell identification, a trained observer manually marked all cells with discernible nuclear contours (based on hematoxylin counterstaining) using the “Cell Counter” plugin, which logged the spatial coordinates of each cell. A cell was scored positive only if it met both objective criteria: 1) the presence of distinct brown-yellow DAB deposition in the nucleus or cytoplasm (visually confirmed); and 2) the optical density (OD) of the staining was quantitatively validated. For this, the “Measure” function was applied to a circular area encompassing the DAB signal of the marked cell. This OD value was then compared to the mean OD value measured from three adjacent, unstained tissue areas within the same field (representing non-specific background); a positive score required the cellular OD to exceed twice this background mean. The positive rate for each compartment was calculated as (Number of positive cells/Total number of cells) × 100%. The final value for each sample was the average of the three fields.

Control experiments: To rigorously validate staining specificity and rule out non-specific background binding, for each immunohistochemical marker, three randomly selected samples were subjected to isotype and negative control procedures. The isotype control used was Rabbit (DA1E) mAb IgG XP® Isotype Control (Cell Signaling Technology, Catalog # 3,900). This control was diluted to the identical protein concentration (µg/mL) as the primary antibody and used under exactly the same incubation conditions (buffer, time, temperature). Negative controls were performed by omitting the primary antibody. Representative images of these controls, confirming the specificity of our staining protocol, are provided in the Supplementary Material (Supplementary Figure S1 for isotype controls; Supplementary Figure S2 for negative controls).

#### 2.3.7 Western blot analysis

Total protein was extracted from homogenized ipsilateral cerebral cortex and full-thickness duodenal tissues. Tissues were homogenized on ice in freshly prepared RIPA lysis buffer supplemented with protease and phosphatase inhibitors. The homogenates were centrifuged at 12,000 × g for 20 min at 4 °C, and the supernatants were collected. Protein concentration was determined using a BCA assay kit (Beyotime, China) with bovine serum albumin (BSA) as the standard. Equal amounts of protein (30 µg per lane) were separated by SDS-PAGE and transferred onto PVDF membranes. Membranes were blocked with 5% non-fat milk and then incubated overnight at 4 °C with primary antibodies against occludin, claudin-5, TLR4, MyD88, NF-κB p65, IL-6, and TNF-α (all 1:1,000). β-Actin (1:5,000) served as a loading control. After washing, membranes were incubated with HRP-conjugated secondary antibodies (1:5,000) for 1 h at room temperature. Protein bands were visualized using an ECL kit and the Bio-Rad Gel Doc XR + system. The grayscale values of protein bands were analyzed using ImageJ software (version 1.53c) with standardized procedures: (i) each lane was analyzed within an identical region of interest (ROI); (ii) local background was subtracted for every band; (iii) target protein signals were normalized to the corresponding β-actin band from the same membrane and exposure. Data are presented as fold change relative to the sham group. All blots were repeated with n = 4 independent biological replicates per group. The raw densitometric values for all key Western blot proteins are provided in Supplementary Table S2.

#### 2.3.8 Serum cytokine measurement

Serum levels of the pro-inflammatory cytokines tumor necrosis factor-alpha (TNF-α) and interleukin-6 (IL-6) were quantified 7 days post-drug administration. Under deep anesthesia, blood was collected from the abdominal aorta. The blood was allowed to clot at room temperature for 30 min and then centrifuged at 3,000 × g for 15 min at 4 °C to separate the serum. The serum supernatant was aliquoted into sterile tubes and stored at −80 °C to prevent cytokine degradation until analysis. Cytokine concentrations were determined using commercial rat-specific enzyme-linked immunosorbent assay (ELISA) kits (Beyotime, China) in accordance with the manufacturer’s protocol. Briefly, standards and serum samples were added in duplicate to the antibody-precoated wells. After incubation and washing as specified, a biotin-conjugated detection antibody was added, followed by incubation with streptavidin-horseradish peroxidase (HRP). Tetramethylbenzidine (TMB) substrate was then added for color development, with incubation at 37 °C for 15 min. The enzymatic reaction was stopped by adding the provided stop solution, and the optical density (OD) of each well was immediately measured at 450 nm using a microplate reader. A standard curve was generated from the OD values of the known standards, and the concentration of TNF-α and IL-6 in each sample was interpolated from this curve. All samples were assayed in duplicate, and results are expressed as picograms per milliliter (pg/mL).

#### 2.3.9 16S rDNA sequencing

The cecal content was collected for 16S rDNA sequencing. It represents the core microbial fermentation site and provides a comprehensive profile of the gut microbiota. Total genomic DNA was extracted from cecal contents using the QIAamp Fast DNA Stool Mini Kit according to the manufacturer’s instructions. The hypervariable V4 region of the bacterial 16S rRNA gene was amplified with primers 515F (GTGCCAGCMGCCGCGGTAA) and 806R (GGACTACHVGGGTWTCTAAT). Amplicons were purified, quantified, and paired-end sequenced (2 × 250 bp) on an Illumina NovaSeq platform. Raw sequencing data were processed using QIIME2 (v2022.8). After quality filtering, denoising, and chimera removal with the DADA2 plugin, amplicon sequence variants (ASVs) were generated. Taxonomy was assigned against the SILVA database (v138). Alpha diversity (within-sample diversity) was calculated using Shannon, Chao1, and Simpson indices. Beta diversity (between-sample diversity) was assessed using Bray-Curtis dissimilarity and visualized via Non-metric Multidimensional Scaling (NMDS) and Principal Coordinate Analysis (PCoA). Permutational multivariate analysis of variance (PERMANOVA) was used to test for significant differences in community structure between groups. Linear discriminant analysis (LDA) effect size (LEfSe) was performed to identify bacterial taxa that were differentially abundant between groups (LDA score >2.0, *p* < 0.05).

### 2.4 Statistical analysis

Data are presented as mean ± standard deviation (SD). Normality was assessed using the Shapiro-Wilk test, and homogeneity of variance was confirmed with Levene’s test. For multi-group comparisons, one-way analysis of variance (ANOVA) was performed, followed by Tukey’s *post hoc* test for normally distributed data. Non-parametric tests were used where appropriate. All statistical analyses were performed using GraphPad Prism software (version 9.0). A *p*-value <0.05 was considered statistically significant.

## 3 Results

### 3.1 Combined AT–PNS treatment provides greater neuroprotection than either monotherapy in MCAO/R rats

**FIGURE 1:**
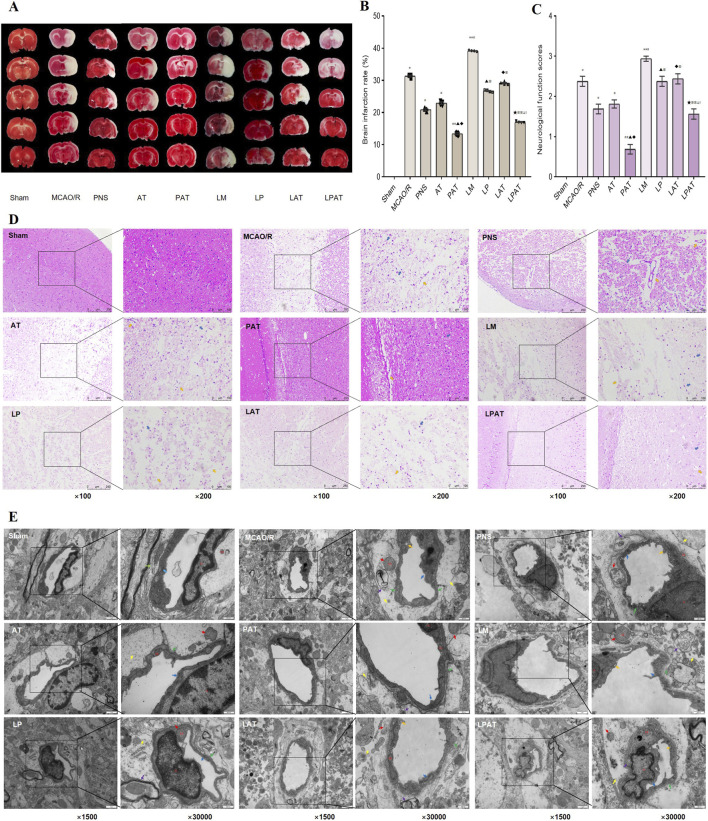
Combined AT–PNS Treatment Provides Greater Neuroprotection Than Either Monotherapy in MCAO/R Rats. **(A)** Representative TTC-stained brain sections. **(B)** Quantitative analysis of cerebral infarction rate (n = 4). **(C)** Neurological function scores. **(D)** Representative light micrographs of rat brain tissue (HE staining). Arrows indicate neuronal pyknosis (blue) and perivascular space widening (yellow). Scale bars: left, 250 μm; right, 100 μm. **(E)** Representative transmission electron microscopy images of rat brain tissue. Arrows indicate: autophagic vacuoles in endothelial cells (purple), disruption of tight junctions (blue), focal dissolution of the basement membrane (green), vacuoles (orange), widened perivascular space (yellow), and mitochondrial swelling (red). Mi, mitochondria; N, nucleus. In the Sham group, arrows denote closed tight junctions and an intact, continuous basement membrane. Scale bars: left, 1 μm; right, 500 nm. Mean ± SD, compared with the Sham group, *p < 0.05, **p < 0.01; compared with the MCAO/R group, ^#^ *p* < 0.05, ^##^ *p* < 0.01; compared with the PNS group, ^▲^ *p* < 0.05; compared with the AT group, ^◆^ *p* < 0.05; compared with the PAT group, ^★^ *p* < 0.05; compared with the LM group, ^⊗^ *p* < 0.05, ^⊗⊗^ *p* < 0.01; compared with the LP group, ^Δ^ *p* < 0.05; compared with the LAT group ^⋄^ *p* < 0.05.

Neuropathological assessments showed that combined AT–PNS treatment produced greater neuroprotective effects than either monotherapy in MCAO/R rats. As shown in Figures 1A–C, rats subjected to MCAO/R exhibited severe neurological deficits and cerebral infarction. While PNS monotherapy moderately improved neurological function and reduced infarct volume, co-administration of AT and PNS (PAT) achieved superior outcomes. To further investigate the therapeutic efficacy under conditions of more severe inflammation simulating post-stroke TLR4 activation, we employed an LPS pre-treatment model. LPS administration significantly exacerbated neurological deficits and cerebral infarction compared to the standard MCAO/R group (Figures 1A–C). Although this LPS challenge attenuated the therapeutic benefits of the drugs, the PAT combination still demonstrated significant protective effects compared to the LPS + MCAO/R (LM) group (*p* < 0.05).

Histopathological analysis (Figure 1D) revealed that structural abnormalities induced by MCAO/R, including perivascular space widening and neuronal vacuolization, were aggravated by LPS. PNS attenuated these pathological changes, with the PAT combination showing enhanced tissue preservation. Ultrastructural examination (Figure 1E) confirmed BBB integrity restoration in PAT groups, showing a reduction in basement membrane dissolution compared to PNS alone. Although LPS compromised therapeutic efficacy, PAT maintained greater protective effects than either monotherapy across the evaluated outcomes.

### 3.2 Combined treatment ameliorates intestinal dysfunction

**FIGURE 2:**
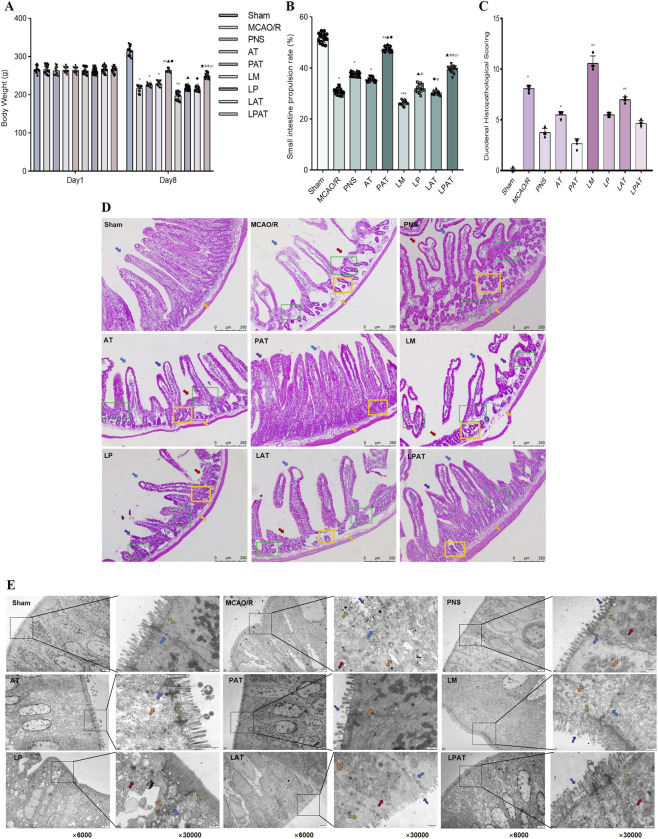
Combined Treatment with AT and PNS Ameliorates MCAO/R-Induced Intestinal Dysfunction. **(A)** Body weight change measured before (Day 1) and after (Day 8) drug administration (n = 16); **(B)** Quantitative assessment of small intestinal propulsion rate (n = 16). **(C)** Histopathological injury scores of duodenal tissues (as shown in D, n = 4, 3 fields per sample). **(D)** Representative light micrographs of duodenal morphology (HE staining). Arrows indicate: epithelium (blue), muscular layer (yellow), epithelial shedding (red), and villus fusion (purple). The green box highlights a representative area of inflammatory infiltration, and the yellow box indicates a representative area of crypt hyperplasia. Scale bar: 250 μm. **(E)** Representative electron micrographs showing ultrastructural changes in duodenal epithelium. Arrows indicate: microvillus shedding (purple), opening of tight junctions (blue), dissolution of the basement membrane (green), mitochondrial swelling (red), and vacuoles (orange). N, nucleus; Mi, mitochondria; RER, rough endoplasmic reticulum; GB, Golgi apparatus. In the Sham group, arrows denote closed tight junctions and an intact, continuous basement membrane. Scale bars: left, 2 μm; right, 500 nm. Mean ± SD, compared with the Sham group, *p < 0.05, **p < 0.01; compared with the MCAO/R group, ^#^ *p <* 0.05, ^##^ *p <* 0.01; compared with the PNS group, ^▲^ *p <* 0.05; compared with the AT group, ^◆^ *p <* 0.05; compared with the PAT group, ^★^ *p <* 0.05; compared with the LM group, ^⊗^ *p <* 0.05, ^⊗⊗^ *p <* 0.01; compared with the LP group, ^Δ^ *p <* 0.05; compared with the LAT group ^⋄^ *p <* 0.05.

A comprehensive evaluation of intestinal injury was conducted at the duodenum, a site highly susceptible to early ischemic damage. The effects of PNS and AT, alone and in combination, were systematically assessed through functional, histological, and ultrastructural analyses (Figure 2).

Rats subjected to MCAO/R exhibited significant intestinal dysfunction, characterized by body weight loss and a markedly decreased small intestinal propulsion rate (Figures 2A,B). Quantitative histopathological scoring confirmed severe duodenal mucosal injury in the model group compared to sham controls (*p* < 0.05; Figure 2C). Representative micrographs revealed characteristic structural damage, including villus fusion, epithelial detachment, and inflammatory cell infiltration in the submucosa (Figure 2D). Ultrastructural examination further identified disruption of epithelial tight junctions and organelle damage, such as mitochondrial swelling (Figure 2E).

Intervention with PNS monotherapy partially alleviated these functional and structural impairments. However, co-administration of PNS and AT (PAT) produced greater restorative effects than either monotherapy, as reflected by more pronounced improvements in intestinal propulsion, mucosal architecture, and ultrastructural integrity.

To evaluate therapeutic resilience under a heightened inflammatory state, an LPS pre-treatment model was employed. LPS administration significantly exacerbated MCAO/R-induced intestinal dysfunction and histopathological damage (Figures 2A–E). Although the therapeutic efficacy of all drugs was attenuated under this pro-inflammatory challenge, the PAT combination consistently maintained superior protective effects compared to the corresponding monotherapies in LPS-pretreated rats (*p* < 0.05), indicating that the combined treatment retained greater protective efficacy under LPS-pretreated conditions.

### 3.3 The AT–PNS combination treatment ameliorates barrier damage

**FIGURE 3:**
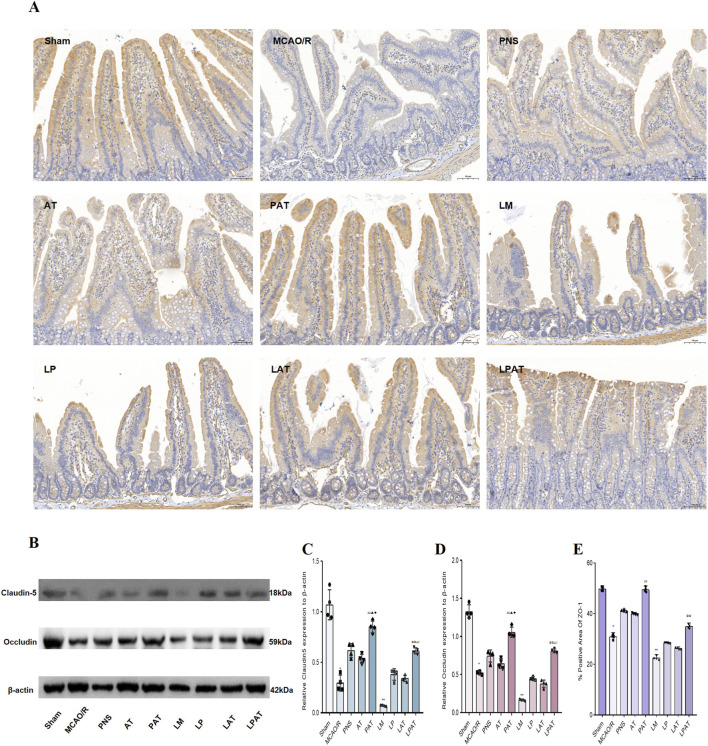
Combined AT–PNS Treatment Restores Intestinal Barrier Integrity After MCAO/R Injury. **(A)** Representative images of ZO-1 immunohistochemistry in duodenal mucosa (scale bar: 100 μm). **(B–D)** Western blot analysis and quantification of Claudin-5 and occludin protein expression in duodenal tissue (n = 4). **(E)** Quantitative analysis of ZO-1 positive immunoreactivity area in duodenal mucosa (as shown in A, n = 4, 3 fields per sample). Mean ± SD, compared with the Sham group, *p < 0.05, **p < 0.01; compared with the MCAO/R group, ^#^ *p <* 0.05, ^##^ *p <* 0.01; compared with the PNS group, ^▲^ *p <* 0.05; compared with the AT group, ^◆^ *p <* 0.05; compared with the LM group, ^⊗^ *p <* 0.05, ^⊗⊗^ *p <* 0.01; compared with the LP group, ^Δ^ *p <* 0.05; compared with the LAT group ^⋄^ *p <* 0.05.

This study specifically investigated the protective effect of the AT–PNS combination on intestinal barrier integrity following MCAO/R injury. Immunohistochemical analysis of the tight junction protein ZO-1 revealed its characteristic honeycomb patterning with robust immunoreactivity in the Sham group (Figure 3A). In contrast, MCAO/R injury led to a fragmented distribution and reduced apical expression intensity of ZO-1. This damage was further exacerbated by LPS pre-treatment (LM group). Co-administration of PNS and AT (PAT) more effectively ameliorated this disruption, as reflected by significantly restored ZO-1 expression compared with the MCAO/R group (*p* < 0.05). The quantitative analysis of ZO-1 immunohistochemical expression is presented in Figure 3E.

Western blot quantification corroborated these findings at the molecular level (Figures 3B–D). The expression levels of the key tight junction proteins claudin-5 and occludin were significantly elevated in the PAT group compared to the MCAO/R model group. Under the pro-inflammatory challenge induced by LPS pre-treatment, the restoration of tight junction proteins was compromised; however, the PAT combination maintained significantly higher expression levels than either PNS or AT monotherapy (*p* < 0.05), suggesting that the combined treatment retained barrier-protective effects under inflammatory challenge.

### 3.4 Combined AT–PNS treatment more strongly suppresses the TLR4/MyD88/NF-κB pathway and downstream inflammatory mediators

**FIGURE 4:**
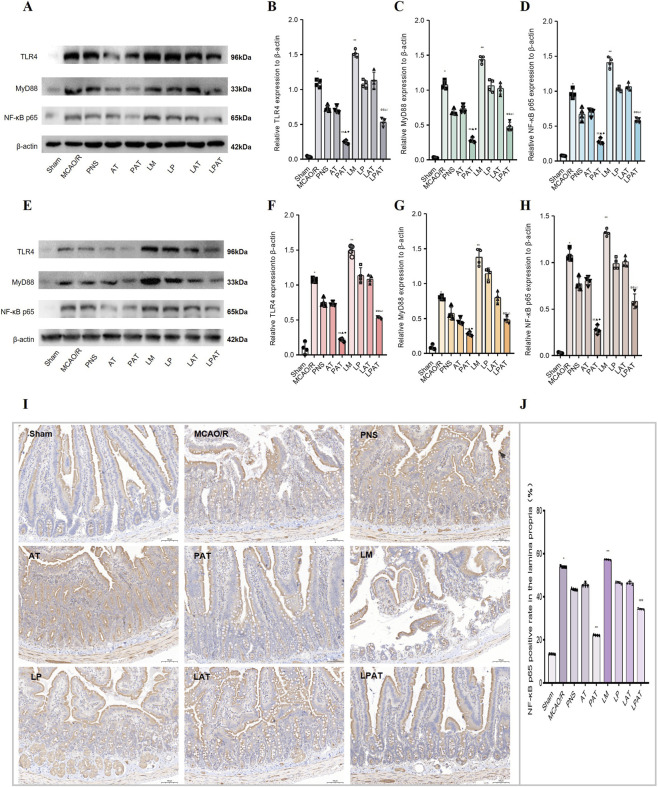
The AT–PNS Combination Suppresses the TLR4/MyD88/NF-κB Signaling Pathway in Brain and Gut Tissues. **(A–D)** Western blot analysis and quantification of TLR4, MyD88, and NF-κB p65 protein expression in brain tissues (n = 4); **(E–H)** Western blot analysis and quantification of TLR4, MyD88, and NF-κB p65 protein expression in duodenal tissues (n = 4); **(I)** Representative immunohistochemical images showing NF-κB p65 expression in duodenal tissues (scale bar: 100 μm); **(J)** Quantification of the NF-κB p65 positive rate in the duodenal lamina propria as shown in panel **(I)** (n = 4, 3 fields per sample). Mean ± SD, compared with the Sham group, *p < 0.05, **p < 0.01; compared with the MCAO/R group, ^#^ *p <* 0.05, ^##^ *p <* 0.01; compared with the PNS group, ^▲^ *p <* 0.05; compared with the AT group, ^◆^ *p <* 0.05; compared with the LM group, ^⊗^ *p <* 0.05, ^⊗⊗^ *p <* 0.01; compared with the LP group, ^Δ^ *p <* 0.05; compared with the LAT group ^⋄^ *p <* 0.05.

We assessed the activation of the TLR4/MyD88/NF-κB signaling pathway, a master regulator of post-stroke inflammation, in both brain and duodenal tissues following MCAO/R. Western blot analysis revealed that MCAO/R injury significantly upregulated the protein expression of TLR4, MyD88, and NF-κB p65 in both tissues (Figures 4A–H). In parallel, immunohistochemistry confirmed a pronounced increase in overall NF-κB p65 expression within the duodenal mucosa of MCAO/R rats (Figure 4I). Pharmacological interventions showed that PNS monotherapy effectively suppressed this pathway activation. Notably, the combination of PNS and AT (PAT) produced a greater suppressive effect than either monotherapy, as reflected by a more pronounced reduction in TLR4, MyD88, and NF-κB p65 expression. In an LPS-pre-treated model used to establish a heightened inflammatory background, the expression of pathway components was further elevated. Although the therapeutic efficacy of all drug regimens was attenuated under this severe challenge, the PAT combination maintained a greater suppressive effect on TLR4/MyD88/NF-κB pathway-related protein expression than either monotherapy.

To delineate the spatial pattern of NF-κB activation within the duodenal mucosa and its link to local inflammation, we quantified NF-κB p65 immunoreactivity in the lamina propria, the primary site of immune cell infiltration (Figure 4J). In the MCAO/R group, NF-κB p65 expression was significantly increased in the lamina propria compared to the Sham group (*p* < 0.05). PAT treatment significantly reduced NF-κB p65 levels in this region (*p* < 0.05 vs. MCAO/R). The NF-κB p65 staining pattern observed in the intestinal epithelium was atypical, showing apical restriction without discernible nuclear staining under any condition. This pattern does not reflect the canonical nuclear translocation indicative of pathway activation in epithelial cells and likely represents non-specific staining. Therefore, we have focused our analysis and interpretation on the lamina propria. The reduction in NF-κB activation within the lamina propria was accompanied by a decrease in inflammatory cell infiltration, supporting an association between pathway attenuation and amelioration of tissue inflammation.

**FIGURE 5:**
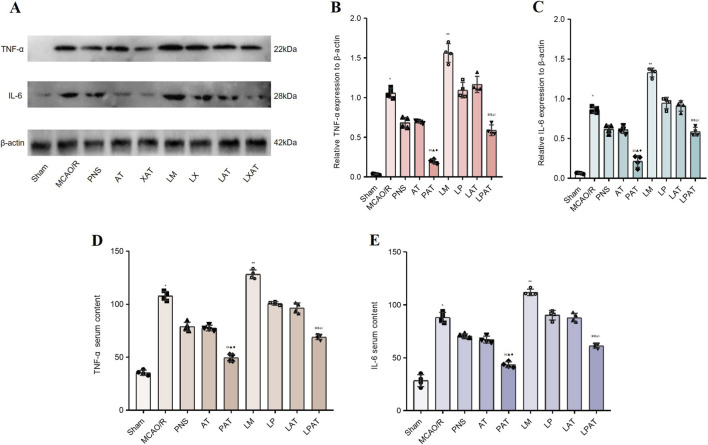
The AT–PNS Combination Attenuates Local and Systemic Inflammation. **(A–C)** Western blot analysis and quantification of TNF-α, IL-6 protein expression in duodenal tissues (n = 4); **(D,E)** Serum concentrations of TNF-α and IL-6 (n = 4). Mean ± SD, compared with the Sham group, ^*^ *p* < 0.05, ^**^ *p <* 0.01; compared with the MCAO/R group, ^#^ *p <* 0.05, ^##^ *p <* 0.01; compared with the PNS group, ^▲^ *p <* 0.05; compared with the AT group, ^◆^ *p <* 0.05; compared with the LM group, ^⊗^ *p <* 0.05, ^⊗⊗^ *p <* 0.01; compared with the LP group, ^Δ^ *p <* 0.05; compared with the LAT group ^⋄^ *p <* 0.05.

Subsequently, we examined the levels of pivotal pro-inflammatory cytokines downstream of the NF-κB pathway. Consistent with the upstream signaling activation, both the duodenal protein expression (Figures 5A–C) and serum concentrations (Figures 5D,E) of TNF-α and IL-6 were significantly elevated after MCAO/R injury. While PNS treatment alone moderately reduced these cytokine levels, the PAT combination exerted a significantly greater suppressive effect. Under the LPS-aggravated condition, cytokine release was further amplified. Nevertheless, the anti-inflammatory effect of the PAT combination remained greater than that of either monotherapy, indicating that the combined treatment retained measurable efficacy in mitigating the inflammatory cascade under LPS-aggravated conditions.

### 3.5 Combined AT–PNS treatment is associated with partial normalization of gut microbiota composition

To investigate whether the greater therapeutic effects of the combined treatment were associated with changes in the gut microbial ecosystem, we performed 16 S rDNA amplicon sequencing on cecal contents, the major site of microbial fermentation.

**FIGURE 6:**
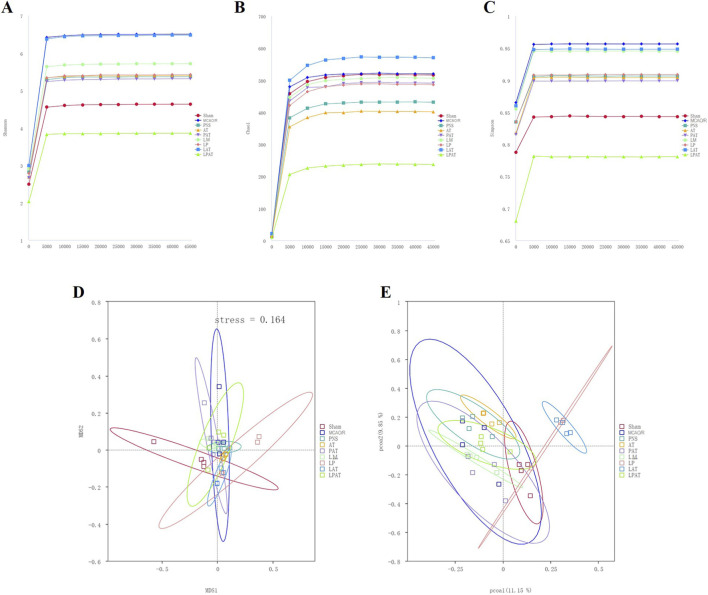
The AT–PNS Combination Is Associated With Changes in Gut Microbial Diversity. **(A–C)** α-Diversity indices assessing microbial richness and evenness (**(A)** Shannon, **(B)** Chao1, **(C)** Simpson); **(D,E)** β-Diversity analyses demonstrating compositional differences between microbial communities (**(D)** NMDS, **(E)** PCoA).

Adequate sequencing depth was confirmed by plateaued rarefaction curves. α-Diversity analysis (Shannon, Chao1, and Simpson indices; Figures 6A–C) demonstrated that the overall gut microbial diversity was significantly compromised by MCAO/R injury. Moreover, distinct β-diversity clustering patterns among groups were revealed by NMDS and PCoA analyses (Figures 6D,E), confirming significant intergroup differences in microbial community structure.

**FIGURE 7:**
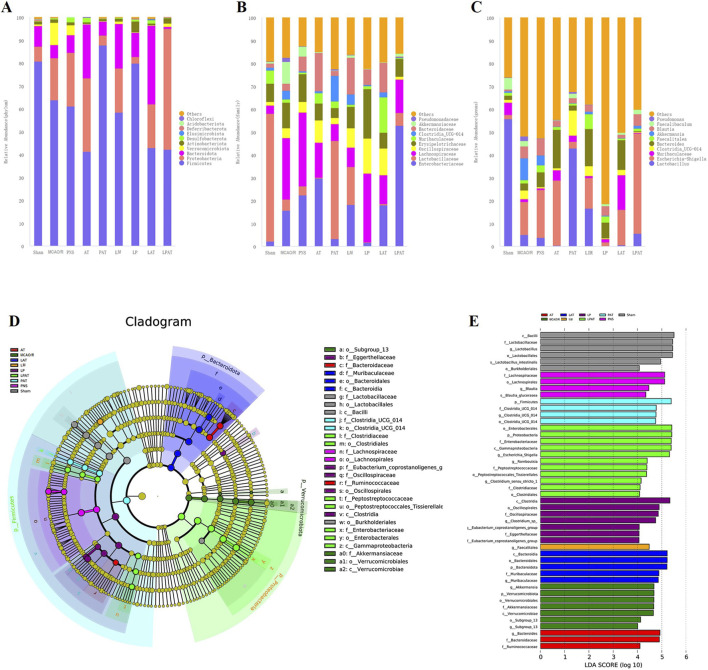
The AT–PNS Combination Is Associated With Altered Gut Microbial Composition at Multiple Taxonomic Levels. **(A–C)** Relative abundance of bacterial taxa at the phylum, family, and genus levels (n = 4). **(D)** Cladogram illustrating the evolutionary relationships of differentially abundant taxa. **(E)** Linear discriminant analysis (LDA) effect size (LEfSe) identifying taxa with statistically significant abundance differences between groups (n = 4).

At the phylum level (Figure 7A), Firmicutes, Bacteroidota, Proteobacteria, and Verrucomicrobiota constituted over 95% of the sequences in Sham controls. The MCAO/R group exhibited a marked dysbiosis, characterized by significant increases in the relative abundances of Proteobacteria and Verrucomicrobiota. Treatment with the PAT combination, but not with either monotherapy alone, significantly restored the Firmicutes/Bacteroidota (F/B) ratio towards sham levels (*p* < 0.05), suggesting that the combined treatment had a stronger association with partial normalization of the core microbial structure than either monotherapy.

Subsequent analyses at finer taxonomic resolutions (Figures 7B,C) identified specific beneficial and pathogenic taxa affected by the treatments. MCAO/R injury led to a depletion of beneficial Lactobacillaceae and an overgrowth of opportunistic pathogens such as Escherichia-Shigella. The PAT intervention partially reversed this imbalance more effectively than either monotherapy, significantly enriching *Lactobacillus* and suppressing pathogenic Escherichia-Shigella compared to the model group (*p* < 0.05).

LEfSe analysis identified group-specific microbial biomarkers (Figures 7D,E). The Sham group was characterized by an abundance of Lactobacillales, whereas the MCAO/R group was distinctly enriched with Verrucomicrobiota and Akkermansia. Notably, the AT and LAT (LPS + AT) groups were uniquely associated with the enrichment of Ruminococcaceae and *Bacteroides*, indicating that AT monotherapy and AT–PNS treatment under inflammatory stress were associated with changes in potentially relevant microbial taxa within the brain-gut axis.

Taken together, the 16S rDNA sequencing data indicate that combined AT–PNS treatment was associated with partial normalization of MCAO/R-induced gut microbial dysbiosis. These compositional changes occurred in parallel with improved barrier integrity and reduced TLR4/MyD88/NF-κB pathway activation. However, because microbial metabolites, circulating LPS, fecal microbiota transplantation, antibiotic depletion, or TLR4 loss-of-function experiments were not performed, the present data do not establish that microbiota changes causally mediate the neurological or anti-inflammatory effects of the treatment.

### 3.6 Quantitative assessment of therapeutic efficacy under LPS-Induced inflammatory challenge

To directly address the impact of systemic inflammation on therapeutic efficacy, we quantified the percentage improvement of key outcome measures for each treatment (PNS, AT, PAT) relative to their respective disease controls (MCAO/R for the non-LPS condition; LPS + MCAO/R for the LPS-pretreated condition), as summarized in Table 1. This analysis revealed that LPS pretreatment differentially modulated the therapeutic landscape. While the absolute protective effects were attenuated under the inflammatory challenge, the relative degree of this attenuation varied markedly across physiological domains and treatment regimens.

**TABLE 1:** Quantitative assessment of therapeutic efficacy and attenuation under LPS challenge.

Evaluation parameter	Treatment group	Improvement vs. MCAO/R (%) (Non-LPS)	Improvement vs. LPS + MCAO/R (%) (LPS-Pretreated)	Efficacy attenuation under LPS (percentage point change)
Neurological function (longa score)	PNS	33.49	31.80	1.69
	AT	26.84	25.64	1.20
	PAT	57.21	56.52	0.69
Cerebral infarct volume	PNS	28.95	19.15	9.80
	AT	23.68	17.02	6.66
	PAT	71.05	46.81	24.24
Intestinal propulsion rate	PNS	22.43	20.90	1.53
	AT	15.30	14.87	0.43
	PAT	54.33	49.85	4.48
Duodenal ZO-1 expression	PNS	33.28	26.70	6.58
	AT	29.15	16.80	12.35
	PAT	60.18	55.13	5.05
Brain TLR4 pathway activation	PNS	30.47	26.96	3.51
	AT	27.16	24.54	2.62
	PAT	70.92	58.59	12.33
Duodenal TLR4 pathway activation	PNS	28.53	25.17	3.36
	AT	25.36	23.69	1.66
	PAT	74.24	55.88	18.36

Notably, for neurological function (Longa score), the relative efficacy of the PAT combination was largely preserved, showing a minimal reduction in percentage improvement of only 0.69 percentage points (from 57.21% to 56.52%). In contrast, the improvement in cerebral infarct volume exhibited greater susceptibility to LPS, with the efficacy of PAT reduced by 24.24 percentage points. Regarding intestinal parameters, the upregulation of ZO-1 expression and the enhancement of the intestinal propulsion rate by PAT were attenuated by 5.05 and 4.48 percentage points, respectively. Most strikingly, the efficacy of PAT in attenuating TLR4/MyD88/NF-κB pathway activation showed a tissue-specific pattern: its effect in brain tissue was reduced by 12.33 percentage points, whereas its effect in duodenal tissue was compromised to a greater extent, with an 18.36 percentage-point reduction. These quantified data underscore the complex and differential impact of pre-existing inflammation on the therapeutic intervention across the brain-gut axis, while suggesting that the combined treatment retained measurable efficacy under heightened inflammatory stress.

## 4 Discussion

### 4.1 The AT–PNS combination confers combinatorial benefit on brain-gut axis function

The brain-gut axis (BGA) is a sophisticated bidirectional communication network integrating neural, endocrine, and immune functions to maintain systemic homeostasis (Cryan et al., 2019). The gut microbiota, through metabolites such as short-chain fatty acids, influences brain function and behavior, forming what is now widely referred to as the microbiota-gut-brain axis (Rutsch et al., 2020). Post-stroke dysregulation of this axis exacerbates intestinal barrier failure and systemic inflammation, contributing to worsened neurological prognosis (Chidambaram et al., 2022). Within this framework, the present study demonstrates that combined administration of *Acorus tatarinowii* Schott (AT) extract and *Panax notoginseng* saponins (PNS) confers a combinatorial benefit that is superior to either monotherapy across multiple endpoints relevant to MCAO/R-induced brain-gut axis dysfunction — including neurological deficits, cerebral infarct volume, blood-brain barrier integrity, intestinal barrier function, and the activation status of the TLR4/MyD88/NF-κB pathway.

It is important at the outset to define precisely what our experimental design can and cannot establish regarding the nature of this combinatorial benefit. The current study employed a single fixed-dose combination (AT 1.56 g/kg/day; PNS 20.8 mg/kg/day) compared against the corresponding monotherapies and disease controls. This design permits demonstration that the combined regimen produces effects greater than either single agent administered alone, but it does not, by itself, formally characterize the pharmacological interaction between AT and PNS, because a full dose-response matrix analyzed by standard methods such as isobolographic analysis or the Chou-Talalay combination index was not generated. We therefore describe our findings as evidence of combinatorial benefit and enhanced therapeutic efficacy warranting further quantitative investigation.

Taken together, the evidence presented in this study supports the conclusion that combined administration of AT and PNS produced greater therapeutic effects on the brain–gut axis than either monotherapy in this fixed-dose design, but a formal pharmacological interaction profile cannot be established without systematic dose–response interaction analysis.

### 4.2 Suppression of TLR4/MyD88/NF-κB signaling is associated with the enhanced brain–gut protective effect of combined AT–PNS treatment

The intestinal barrier, composed of epithelial cells and tight junctions, is critical for preventing bacterial translocation and maintaining intestinal homeostasis (Saitoh et al., 2015; Díaz-Coránguez et al., 2019). Tight junction proteins, such as ZO-1, occludin, and claudin-5, are fundamental to barrier integrity (Furuse, 2010). In the present study, MCAO/R caused obvious intestinal barrier injury, as reflected by reduced tight junction protein expression, villus disruption, epithelial damage, inflammatory cell infiltration, and ultrastructural abnormalities. Combined AT–PNS treatment was associated with more evident restoration of tight junction protein expression and mucosal structure than either AT or PNS monotherapy.

The TLR4/MyD88/NF-κB signaling pathway is closely related to inflammatory responses after cerebral ischemia-reperfusion injury. Cerebral ischemia can activate TLR4 through damage-associated molecular patterns and gut-derived inflammatory stimuli, leading to MyD88-dependent NF-κB activation and increased production of pro-inflammatory cytokines such as TNF-α and IL-6 (Lim and Staudt, 2013; Leitner et al., 2019). This inflammatory cascade may further aggravate barrier injury and contribute to a pathological feedback loop between the brain and gut (Zhao et al., 2014; Zhao et al., 2018). In our study, MCAO/R increased the expression of TLR4, MyD88, and NF-κB p65 in both brain and duodenal tissues, together with elevated TNF-α and IL-6 levels. Compared with either monotherapy, combined AT–PNS treatment produced a greater reduction in TLR4/MyD88/NF-κB pathway-related proteins and inflammatory cytokines, suggesting that attenuation of this pathway may be associated with its enhanced brain–gut protective effect.

The LPS-pretreated model was used to assess treatment effects under an aggravated inflammatory condition. LPS, as a canonical TLR4 agonist, further enhanced inflammatory pathway activation and worsened brain–gut axis injury. Although the protective effects of all treatments were partly attenuated under LPS challenge, combined AT–PNS treatment still showed greater attenuation of TLR4/MyD88/NF-κB signaling and downstream cytokine expression than either monotherapy. This result suggests that the combined treatment retains anti-inflammatory effects under heightened inflammatory stress, but it should not be interpreted as evidence of direct TLR4 antagonism.

Taken together, these findings indicate that combined AT–PNS treatment attenuates MCAO/R-induced inflammatory activation in both the brain and gut more effectively than either monotherapy. Attenuation of TLR4/MyD88/NF-κB signaling is therefore an inflammatory pathway associated with the observed brain–gut protective effects. Nevertheless, because this study did not include direct target-binding experiments, TLR4 loss-of-function validation, or formal dose-response interaction analysis, these findings should be interpreted as evidence of pathway-associated anti-inflammatory effects rather than definitive proof of direct pharmacological TLR4 inhibition or a formal dose-response interaction profile.

### 4.3 Gut microbiota changes associated with AT–PNS treatment: a biologically plausible but currently unproven link to TLR4 signaling

A central question raised by our 16S rDNA sequencing data is whether — and how — the observed changes in gut microbial composition relate mechanistically to the inhibition of the TLR4/MyD88/NF-κB pathway documented elsewhere in this study. We address this question with explicit attention to what our data can, and cannot, establish.

#### 4.3.1 What we observed

MCAO/R injury was accompanied by a marked dysbiotic shift in the cecal microbiota, characterized by reduced α-diversity, a decreased Firmicutes/Bacteroidota ratio, expansion of Gram-negative Proteobacteria (notably Escherichia-Shigella), enrichment of Verrucomicrobiota/Akkermansia, and depletion of beneficial commensals including Lactobacillaceae and members of Lachnospiraceae/Ruminococcaceae (Figures 6, 7). Among all treatment groups, only the AT–PNS combination (PAT) was associated with a substantial partial restoration of these features — increasing the Firmicutes/Bacteroidota ratio, reducing Proteobacteria abundance, and enriching *Lactobacillus* and short-chain fatty acid (SCFA)-producing taxa — toward, though not fully reaching, sham-control levels. In parallel, the same treatment group exhibited the most pronounced suppression of TLR4, MyD88, and NF-κB p65 expression in both brain and duodenal tissues, the greatest reduction in pro-inflammatory cytokines (TNF-α, IL-6), and the most complete restoration of barrier integrity (Figures 3–5).

These two sets of observations, microbiota normalization and TLR4 pathway suppression, co-occurred consistently across the same treatment condition, both at baseline and under LPS challenge. This co-occurrence raises the question of whether they are mechanistically connected, and if so, in what direction.

#### 4.3.2 Biologically plausible mechanistic links between gut microbiota and TLR4/MyD88/NF-κB signaling

A substantial body of literature describes well-characterized molecular routes by which compositional changes in the gut microbiota can modulate TLR4-dependent inflammatory signaling (Zhu et al., 2024). Three such routes are most directly relevant to the taxonomic shifts observed in our study:

- LPS as the canonical endogenous TLR4 ligand. Lipopolysaccharide, derived primarily from the outer membrane of Gram-negative bacteria, is the prototypical agonist of the TLR4/MD2 complex. Expansion of Gram-negative pathobionts (such as the Escherichia-Shigella enrichment observed in our MCAO/R group) increases the luminal pool of LPS available for translocation across a compromised intestinal barrier into the portal and systemic circulation — a phenomenon termed “metabolic endotoxemia” (Li et al., 2025). The PAT-associated reduction in Proteobacteria abundance is, in principle, consistent with a reduced systemic LPS burden and, by extension, reduced tonic activation of TLR4 in both gut-resident and brain-resident immune cells. We emphasize, however, that we did not measure circulating or luminal LPS levels in the present study, and therefore cannot directly attribute the observed TLR4 pathway suppression to a reduction in LPS load.
- Short-chain fatty acids as endogenous suppressors of NF-κB activation. Butyrate, propionate, and acetate, produced by fermentation of dietary fiber by Firmicutes, particularly Lachnospiraceae and Ruminococcaceae, are well-documented inhibitors of NF-κB activation in intestinal epithelial and immune cells, acting via histone deacetylase inhibition and GPR43/GPR109 A signaling (Vacca et al., 2026). The PAT-associated enrichment of SCFA-producing taxa and the partial restoration of the Firmicutes/Bacteroidota ratio could, in principle, increase luminal SCFA availability and contribute to NF-κB suppression downstream of TLR4. As with LPS, however, we did not perform targeted SCFA quantification and cannot directly demonstrate this contribution.
- Barrier-protective commensals reducing PAMP translocation. *Lactobacillus* species and butyrate-producing bacteria reinforce intestinal tight junctions through induction of ZO-1, occludin, and claudin expression, thereby reducing paracellular translocation of microbial pathogen-associated molecular patterns (PAMPs) including LPS (Djukovic et al., 2022; Chen et al., 2022). The PAT-associated enrichment of *Lactobacillus* and the concurrent restoration of duodenal ZO-1, claudin-5, and occludin expression (Figure 3) are mutually consistent with this barrier-reinforcing route, though the direction of causality, whether barrier restoration enables microbial normalization or *vice versa*, cannot be resolved by our current data.

#### 4.3.3 Proposed but unproven mechanistic link

We wish to state explicitly: while the three routes outlined above provide a coherent and biologically plausible framework for interpreting the co-occurrence of microbiota modulation and TLR4 pathway suppression in our data, the present study does not directly demonstrate that the microbiota changes are causally responsible for, or even contribute to, the observed TLR4 pathway inhibition. The 16 S rDNA findings establish an associated ecological shift, not a proven mechanistic driver. Several alternative interpretations are equally compatible with our data:

The microbiota changes may be a consequence rather than a cause of reduced inflammation: by attenuating local and systemic inflammatory signaling, including reduced expression of TLR4/MyD88/NF-κB pathway-related proteins, the AT–PNS combination may create a less inflamed luminal environment that secondarily permits the recovery of oxygen-sensitive commensals and the suppression of facultatively anaerobic pathobionts.

The microbiota and TLR4 changes may be parallel correlates of an upstream pharmacological effect—for example, AT-mediated improvement of intestinal motility and barrier function—without a direct causal link between the two.

The two may form a bidirectional positive-feedback relationship, in which reduced inflammation enables microbiota recovery, and microbiota recovery further reduces inflammatory drive.

Distinguishing among these possibilities is beyond what the present experimental design permits. Definitive resolution would require: (a) direct quantification of luminal and circulating LPS to test the LPS-translocation hypothesis; (b) targeted metabolomics for SCFAs, secondary bile acids, and tryptophan-derived metabolites to characterize the functional output of the modulated microbiota; (c) fecal microbiota transplantation (FMT) experiments transferring PAT-shaped microbiota into naïve MCAO/R recipients to test sufficiency; and (d) antibiotic-depletion or germ-free control studies to test necessity. None of these experiments were performed in the present study, and we have therefore explicitly refrained from claiming a causal microbiota → TLR4 axis in our conclusions.

#### 4.3.4 Why monotherapy did not reproduce the microbiota-modulating effect

A notable observation is that neither PNS nor AT monotherapy significantly restored the gut microbial structure to a comparable degree, despite each producing partial effects on barrier integrity and TLR4 signaling. We do not attempt to explain this finding in terms of a formally characterized pharmacological interaction; for the reasons set out in Section 4.1, our experimental design does not support such a claim. Two non-mutually-exclusive interpretations remain compatible with the data and warrant testing in future work.

- Threshold-dependent ecological recovery. The gut microbial community may exhibit threshold-like dynamics, in which substantial compositional restoration requires the inflammatory drive to fall below a critical level. If neither monotherapy reduces TLR4 activation and downstream cytokine production sufficiently to cross this threshold—but their combination does—the observed pattern would follow naturally, without requiring a direct pharmacological interaction at the level of microbiota modulation itself.
- Complementary pharmacological actions of the two agents. AT and PNS may act on distinct but complementary determinants of the gut microenvironment—for example, AT improving intestinal motility and reducing transit-time-dependent dysbiosis, while PNS reduces systemic inflammatory drive feeding back into the lumen. Either component alone may be insufficient to produce ecologically meaningful restructuring, whereas their combination engages both determinants simultaneously.

These interpretations are explicitly framed as hypotheses to be tested, not as conclusions drawn from the present data. We identify the experimental approaches needed to distinguish them as a key direction for future work (see Section 4.5).

### 4.4 Rationale for LPS pretreatment and analysis of context-dependent drug efficacy

A pivotal objective of this study was to rigorously evaluate the resilience of the PAT combination against a background of heightened systemic inflammation. Clinically, up to 30% of ischemic stroke patients develop stroke-associated infections (SAI), which act as a “second hit” to exacerbate systemic inflammation and worsen neurological outcomes. By employing a low-dose LPS pretreatment model, we utilized a canonical TLR4 ligand not merely as a generic inflammatory stimulus, but as a targeted pharmacological tool to pre-activate the precise TLR4/MyD88/NF-κB pathway that our combination therapy aims to inhibit. This paradigm establishes a stringent “stress-test” to assess whether the therapy retains meaningful efficacy when the pathological inflammatory drive is amplified.

Our quantitative analysis (Table 1) revealed a fascinating tissue-specific divergence in therapeutic attenuation under the LPS challenge. The neuroprotective efficacy of PAT was remarkably preserved (with only a 0.69 percentage point loss in neurological score improvement), whereas its protective effects on the intestinal barrier (e.g., ZO-1 expression) and duodenal TLR4 pathway inhibition experienced more pronounced attenuation (18.36 percentage point reduction). This differential vulnerability likely stems from the distinct anatomical and immunological landscapes of the brain-gut axis. The duodenum, acting as a primary mucosal immune interface with dense populations of resident macrophages and lymphocytes, is directly and massively exposed to systemic endotoxemia, which may partially overwhelm local reparative mechanisms. In contrast, despite ischemic compromise, the blood-brain barrier (BBB) may still offer a degree of compartmentalization. Furthermore, AT is known to enhance BBB permeability for co-administered drugs; thus, the PAT combination may ensure sufficient central bioavailability of PNS to sustain neuroprotection even when peripheral inflammation is rampant.

Crucially, the maintained, albeit variably attenuated, efficacy of PAT across all metrics under the LPS challenge indicates that the combined treatment retained measurable protective effects under heightened inflammatory stress. This pharmacological resilience may be related to the multi-target profile of the combined treatment. By affecting both central inflammatory responses and peripheral gut-related alterations, the PAT combination may offer a broader protective profile under systemic inflammatory stress, although its translational relevance requires further validation in clinically relevant models.

### 4.5 Study limitations and directions for future research

Several important limitations constrain the interpretation of our findings and define the agenda for follow-up work.

First and most fundamentally, the present study employed a single fixed-dose combination of AT and PNS compared with the corresponding monotherapies. This design allows us to demonstrate that the combined regimen produced effects superior to either monotherapy, but it does not formally characterize the pharmacological interaction between the two agents because full dose-response matrices required for isobolographic or Chou–Talalay analysis were not generated. Accordingly, the *post hoc* Bliss-deviation analysis based on the percentage-improvement values in Table 1 (full results in Supplementary Table S3) is presented only as an exploratory descriptive assessment and should not be interpreted as formal pharmacological evidence of synergy, additivity, or a defined drug interaction. Definitive characterization will require future studies using checkerboard dose-response designs analyzed by isobolographic or Chou–Talalay methods.

Second, while our 16 S rDNA sequencing data establish a clear association between gut microbial restructuring and TLR4/MyD88/NF-κB pathway suppression, the present study does not directly demonstrate a causal pharmacological connection between them. As discussed in Section 4.3, our data are equally compatible with the microbiota changes being a cause, a consequence, or a parallel correlate of reduced TLR4 signaling. Resolving this question will require approaches we did not undertake here—including direct quantification of luminal and circulating LPS, targeted metabolomics for SCFAs and other microbial metabolites, fecal microbiota transplantation to test sufficiency, and antibiotic-depletion or TLR4 loss-of-function models to test necessity. We have therefore restricted our conclusions to what the present data can support: an associated ecological shift consistent with a biologically plausible but unproven mechanistic link.

Third, the dose of AT extract administered (1.56 g/kg/day) exceeds the 1 g/kg/day threshold recommended by contemporary phytopharmacological guidelines (Heinrich et al., 2020). This dose was chosen on the basis of traditional use and our preliminary efficacy work, but its direct extrapolation to human equivalents is not pharmacologically meaningful, and the high exposure may introduce non-specific effects. Future work must include systematic dose-response analyses to determine the minimum effective dose.

Fourth, the clinically used formulation of PNS (Xuesaitong Injection) is administered intravenously, whereas we used the intraperitoneal route in this rodent model for consistency with foundational pharmacokinetic studies (Yang and Zhao, 2024). Future preclinical work should evaluate efficacy via a clinically relevant route of administration. We also note that outcomes were assessed at a single early time point in male animals only; validation across longer recovery windows, in female animals, and with identification of the specific bioactive constituents responsible for the observed effects will be essential for translational development.

In summary, the present study demonstrates that combined administration of AT and PNS produces a combinatorial benefit on the brain-gut axis following cerebral ischemia-reperfusion injury, with effects measurably superior to either monotherapy and an associated suppression of TLR4/MyD88/NF-κB signaling and partial restoration of gut microbial composition. Definitive characterization of the AT–PNS interaction and establishment of the microbiota-TLR4 link await the dose-response and causal-mechanistic studies outlined above. We hope this work provides both a therapeutic rationale and a methodological roadmap for the rigorous pharmacological characterization that should follow.

## Data Availability

The original contributions presented in the study are included in the article/Supplementary Material, further inquiries can be directed to the corresponding author.
