# Supplementary material for: Combined treatment with Acorus tatarinowii Schott and Panax notoginseng saponins ameliorates brain–gut axis dysfunction in MCAO/R rats with suppression of TLR4/MyD88/NF-κB signaling and associated gut microbiota changes
Source: Front Pharmacol. 2026 Jun 29;17:1683558. doi: 10.3389/fphar.2026.1683558 (PMC13357153; doi:10.3389/fphar.2026.1683558)
Supplement: Supplementary file 1 [file Table1.docx]

**Supplementary Material Overview**

The main Supplementary Material contains Tables S1–S3 and Figures S1–S2.

A separate Supplementary File S1, entitled “Botanical Drug Characterization and Orthogonal Fingerprinting of *Acorus tatarinowii* Schott Extract,” is provided in the accompanying supplementary package. Tables and figures in Supplementary File S1 are numbered independently as Table SF1-1 to Table SF1-5 and Figure SF1-1 to Figure SF1-3. The corresponding raw/supporting files are provided as Supplementary Data S1A–S1E.

**Tables**

**Table S1 Hepatic and Renal Function Indicators in Normal Rats after 7-Day Administration**

|  | **ALT (U/L)** | **AST (U/L)** | **BUN (mg/dL)** | **CREA (μmol/L)** |
| --- | --- | --- | --- | --- |
| Sham1 | 27.908 | 90.324 | 20.574 | 63.726 |
| Sham2 | 36.355 | 99.653 | 9.673 | 58.403 |
| Sham3 | 36.054 | 78.811 | 22.039 | 52.167 |
| PNS1 | 48.803 | 87.983 | 16.439 | 58.099 |
| PNS2 | 32.102 | 88.353 | 14.174 | 55.057 |
| PNS3 | 39.373 | 86.538 | 9.786 | 24.335 |
| AT1 | 41.332 | 91.376 | 9.878 | 80.000 |
| AT2 | 32.060 | 88.868 | 12.286 | 62.814 |
| AT3 | 37.639 | 93.628 | 12.827 | 63.726 |
| PAT1 | 27.233 | 88.913 | 22.109 | 29.962 |
| PAT2 | 31.927 | 88.610 | 20.832 | 63.422 |
| PAT3 | 38.873 | 80.737 | 21.756 | 52.015 |
| reference value | 21.53-61.75 | 41.47-195.65 | 9.75-22.71 | 10.90-118.07 |

Note: Group definitions: Sham: Administered saline by gavage; AT: Received *Acorus tatarinowii* Schott (AT) aqueous extract at 1.56 g/kg/d by gavage; PNS: Received *Panax notoginseng* saponins (PNS) at 20.8 mg/kg/d intraperitoneally; PAT: Co-administered AT (1.56 g/kg/d, gavage) and PNS (20.8 mg/kg/d, i.p.). Liver Function: ALT: Alanine Aminotransferase; AST: Aspartate Aminotransferase. Renal Function: BUN: Blood Urea Nitrogen, CREA: Creatinine.

**Table S2 The raw densitometric values (mean ± SD) for all key proteins**

|  | **Sham** | **MCAO/R** | **PNS** | **AT** | **PAT** | **LM** | **LP** | **LAT** | **LPAT** |
| --- | --- | --- | --- | --- | --- | --- | --- | --- | --- |
| **Brain TLR4** | 0.04±0.01 | 1.09±0.05 | 0.73±0.04 | 0.71±0.05 | 0.25±0.02 | 1.52±0.05 | 1.08±0.06 | 1.13±0.12 | 0.53±0.06 |
| **Brain MyD88** | 0.03±0.01 | 1.08±0.06 | 0.69±0.04 | 0.73±0.05 | 0.28±0.03 | 1.44±0.05 | 1.07±0.09 | 1.02±0.08 | 0.48±0.06 |
| **Brain NF-κB p65** | 0.08±0.00 | 0.97±0.06 | 0.67±0.09 | 0.71±0.06 | 0.28±0.04 | 1.41±0.08 | 1.03±0.03 | 0.99±0.06 | 0.58±0.03 |
| **duodenum TLR4** | 0.10±0.08 | 1.07±0.03 | 0.75±0.06 | 0.73±0.02 | 0.21±0.02 | 1.50±0.07 | 1.14±0.11 | 1.09±0.06 | 0.53±0.02 |
| **duodenum MyD88** | 0.04±0.01 | 1.00±0.07 | 0.70±0.02 | 0.69±0.04 | 0.30±0.03 | 1.42±0.09 | 1.10±0.06 | 1.09±0.08 | 0.54±0.06 |
| **duodenum NF-κB p65** | 0.03±0.01 | 1.03±0.04 | 0.77±0.07 | 0.81±0.04 | 0.28±0.04 | 1.37±0.05 | 0.99±0.06 | 1.01±0.05 | 0.58±0.08 |
| **claudin5** | 1.07±0.15 | 0.29±0.08 | 0.62±0.07 | 0.53±0.04 | 0.85±0.06 | 0.07±0.01 | 0.38±0.06 | 0.34±0.03 | 0.61±0.03 |
| **Occludin** | 1.33±0.09 | 0.52±0.02 | 0.74±0.08 | 0.64±0.06 | 1.06±0.07 | 0.17±0.01 | 0.45±0.02 | 0.38±0.05 | 0.81±0.02 |
| **IL-6** | 0.06±0.01 | 0.86±0.04 | 0.62±0.05 | 0.61±0.05 | 0.21±0.07 | 1.33±0.06 | 0.95±0.07 | 0.91±0.06 | 0.59±0.05 |
| **TNF-α** | 0.03±0.01 | 1.06±0.06 | 0.68±0.07 | 0.70±0.01 | 0.20±0.02 | 1.56±0.12 | 1.10±0.10 | 1.17±0.10 | 0.59±0.07 |

**Table S3 Exploratory Post-hoc Bliss-Deviation Analysis of the AT–PNS Combination Across Multiple Endpoints**

As an exploratory descriptive analysis, a post-hoc Bliss-deviation analysis was performed using the percentage-improvement values reported in Table 1 of the main manuscript. The expected value was calculated under the Bliss independence assumption. Because the present analysis is based on group-mean improvements from a single fixed-dose combination design rather than full dose–response matrices, it should be interpreted only as a hypothesis-generating assessment and not as formal pharmacological evidence of synergy or a defined drug interaction.

*E*expected *= E*A *+ E*B *− (E*A *× E*B*)*

where EA and EB are the fractional improvements produced by PNS and AT monotherapy, respectively, and Δ was calculated as Eobserved − Eexpected. A positive deviation indicates only that the observed value numerically exceeded the value predicted under the Bliss independence assumption. However, this result does not establish formal pharmacological synergy, because isobolographic or Chou–Talalay analysis based on full dose–response matrices was not performed. For consistency with the reporting style of Table 1 in the main manuscript, all values in the present table are expressed as percentages.

| **Endpoint** | **E_PNS / E_LP (%)** | **E_AT / E_LAT (%)** | **E_expected (%)** | **E_observed (%)** | **Δ (%)** | **Interpretation** |
| --- | --- | --- | --- | --- | --- | --- |
| **Part A. Bliss independence analysis under standard MCAO/R conditions (PAT vs. PNS + AT)** | | | | | | |
| Neurological function (Longa score) | 33.49 | 26.84 | 51.34 | **57.21** | **+5.87** | Observed value exceeded Bliss-predicted value; exploratory only |
| Cerebral infarct volume | 28.95 | 23.68 | 45.77 | **71.05** | **+25.28** | Observed value exceeded Bliss-predicted value; exploratory only |
| Intestinal propulsion rate | 22.43 | 15.30 | 34.30 | **54.33** | **+20.03** | Observed value exceeded Bliss-predicted value; exploratory only |
| Duodenal ZO-1 expression | 33.28 | 29.15 | 52.73 | **60.18** | **+7.45** | Observed value exceeded Bliss-predicted value; exploratory only |
| Brain TLR4 pathway suppression | 30.47 | 27.16 | 49.35 | **70.92** | **+21.57** | Observed value exceeded Bliss-predicted value; exploratory only |
| Duodenal TLR4 pathway suppression | 28.53 | 25.36 | 46.66 | **74.24** | **+27.58** | Observed value exceeded Bliss-predicted value; exploratory only |
| **Part B. Bliss independence analysis under LPS-pretreated conditions (LPAT vs. LP + LAT)** | | | | | | |
| Neurological function (Longa score) | 31.80 | 25.64 | 49.29 | **56.52** | **+7.23** | Observed value exceeded Bliss-predicted value; exploratory only |
| Cerebral infarct volume | 19.15 | 17.02 | 32.91 | **46.81** | **+13.90** | Observed value exceeded Bliss-predicted value; exploratory only |
| Intestinal propulsion rate | 20.90 | 14.87 | 32.66 | **49.85** | **+17.19** | Observed value exceeded Bliss-predicted value; exploratory only |
| Duodenal ZO-1 expression | 26.70 | 16.80 | 39.01 | **55.13** | **+16.12** | Observed value exceeded Bliss-predicted value; exploratory only |
| Brain TLR4 pathway suppression | 26.96 | 24.54 | 44.88 | **58.59** | **+13.71** | Observed value exceeded Bliss-predicted value; exploratory only |
| Duodenal TLR4 pathway suppression | 25.17 | 23.69 | 42.90 | **55.88** | **+12.98** | Observed value exceeded Bliss-predicted value; exploratory only |

**Notes and interpretation:**

**(1)** Under standard MCAO/R conditions (Part A), the observed values for the PAT regimen numerically exceeded the Bliss-predicted values across all six endpoints assessed (Δ ranging from +5.87% to +27.58%). The largest positive numerical deviations were observed for duodenal TLR4 pathway suppression (+27.58%), cerebral infarct volume reduction (+25.28%), and brain TLR4 pathway suppression (+21.57%). These findings are descriptive and exploratory only, and they should not be interpreted as evidence of formal pharmacological synergy or a defined drug interaction.

(2) Under LPS-pretreated challenge conditions (Part B), positive numerical Bliss deviations were observed across all six endpoints (Δ ranging from +7.23% to +17.19%). These data indicate that the observed PAT values remained numerically higher than the Bliss-predicted values under heightened inflammatory stress, although the magnitude of the deviation was generally attenuated relative to the non-LPS condition. This exploratory comparison should not be interpreted as evidence of formal pharmacological synergy.

**(3)** Interpretive caution. The Bliss independence model assumes mechanistic independence between the two agents, an assumption that may not strictly hold for AT and PNS, which appear to converge on overlapping pathways (TLR4/MyD88/NF-κB signaling, barrier protection, microbiota-associated changes). Furthermore, the present analysis is based on group-mean improvements derived from a single fixed-dose combination design, and therefore *cannot substitute for a formal isobologram or Chou-Talalay analysis derived from full dose-response matrices*. Accordingly, the results presented here are interpreted only as hypothesis-generating numerical deviations from Bliss-predicted values, rather than as evidence of formal pharmacological synergy, additivity, or a defined drug interaction.

**(4)** Future validation requirement. Definitive characterization of the AT–PNS interaction will require future studies employing systematic dose-response matrices — for example, a checkerboard design covering multiple doses of AT and PNS, both individually and in combination — analyzed by rigorous methods such as isobolographic analysis (Loewe additivity), the Chou-Talalay combination index (CI), or response-surface modeling. Such work is explicitly identified as a priority for the next phase of this research program.

**Abbreviations:**

AT, Acorus tatarinowii Schott aqueous extract; PNS, Panax notoginseng saponins; PAT, combination of PNS and AT; LM, lipopolysaccharide (LPS) + MCAO/R; LP, LPS + PNS; LAT, LPS + AT; LPAT, LPS + PAT; MCAO/R, transient middle cerebral artery occlusion/reperfusion; TLR4, toll-like receptor 4; ZO-1, zonula occludens-1; Δ, Bliss deviation (E_observed − E_expected).

**Figures**

**
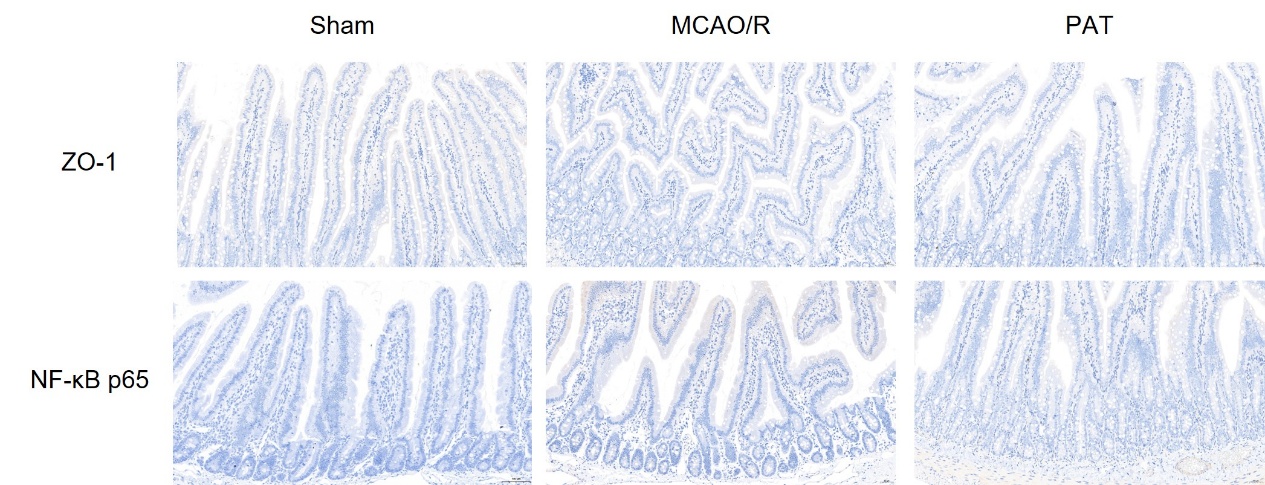
**

**Figure S1. Isotype control images for ZO-1 and NF-κB p65 immunohistochemistry.**


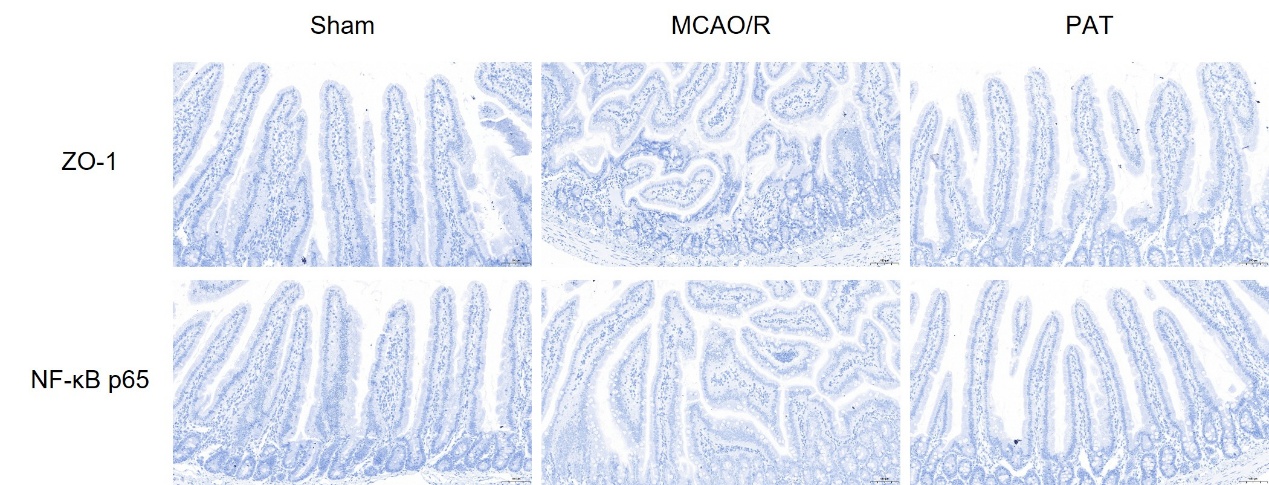


**Figure S2. Negative control images for ZO-1 and NF-κB p65 immunohistochemistry.**
