# Supplementary material for: Combined treatment with Acorus tatarinowii Schott and Panax notoginseng saponins ameliorates brain–gut axis dysfunction in MCAO/R rats with suppression of TLR4/MyD88/NF-κB signaling and associated gut microbiota changes
Source: Front Pharmacol. 2026 Jun 29;17:1683558. doi: 10.3389/fphar.2026.1683558 (PMC13357153; doi:10.3389/fphar.2026.1683558)
Supplement: Supplementary file 2 [file DataSheet1.zip › Supplementary_Materials/Supplementary_Data_S1A_Q-Orbitrap_HRLCMS_Report.pdf]

基于 Q-Orbitrap 高分辨液质联用对中药液中化学成分的鉴定研究报告

研究单位：武汉赛维尔生物科技有限公司

研究单位地址：湖北省武汉市东湖高新技术开发区佛祖岭二路 4 号武汉赛维尔生物科技有限公司

委托人： 郝琳瑶

项目负责人：刘瑶

项目开始日期：2026.01.14

项目结束日期：2026.02.10

1 介绍

小分子类化合物经高分辨质谱分析采集数据（MS1，MS2），最后通过相关的软件解析这些质谱图就可以得到样品中化合物定性鉴定信息。

2 项目信息

2.1 项目目标

本项目拟通过质谱手段对中药液中的成分进行质谱解析鉴定。

2.2 样品信息

中药液 1 份。

3 材料设备与方法

甲醇：色谱纯，赛默飞世尔科技（中国）有限公司

甲酸：色谱纯，上海阿拉丁生化科技股份有限公司

聚丙烯离心管：1.5mL，2.0mL，武汉赛维尔生物科技有限公司

纯水/超纯水一体机系统：Direct-Q®5，德国默克密理博

速微量冷冻离心机：D3024R，北京大龙兴创实验仪器有限公司

涡旋振荡器：MX-F，武汉赛维尔生物科技有限公司

超声波清洗器：JP-040S，深圳洁盟清洗设备有限公司

移液器：2.0~20.0 μL，20.0~200 μL，200~1000 μL， Eppendorf

色谱仪：UltiMate 3000 RS，赛默飞世尔科技（中国）有限公司

质谱仪：Q Exactive 高分辨质谱仪， 赛默飞世尔科技（中国）有限公司

3.1 样品处理

取中药液 200μL，加入 800 μL 甲醇，和研磨珠，研磨 5 min，涡旋混匀 10 min，13000 rpm 离心 10 min，取上清上机分析。

3.2 检测条件

3.2.1 质谱条件

离子源：电喷雾电离源（ESI）

扫描方式：正负离子切换扫描

检测方式：Full mass/dd-MS2

分辨率：70000（full mass）；17500（dd-MS2）

扫描范围（scan range）：100.0～1500.0 m/z

电喷雾电压（Spary Voltage）：3.2 kV（Positive, Negative）

毛细管温度（Capillary Temperature）：300 ℃

碰撞气：高纯氩气（纯度≥99.999 %）

碰撞能(N)CE：30，40，60

鞘气：氮气（纯度≥99.999%），40 Arb

辅助气：氮气（纯度≥99.999%），15 Arb ，350 ℃

数据采集时间：80.0 min

3.2.2 色谱条件

色谱柱：Welch Ultimate Plus-C18 4.6×250 mm, 5 μm

流速：0.80 mL/min

水相：0.1 %甲酸/水溶液

有机相：乙腈

柱温箱温度：35℃

自动进样器温度：10.0℃

自动进样器进样体积：5.00 μL

Table1 色谱梯度

时间（min）	水相比例（%）	有机相比例（%）
10	99	1
20	70	30
30	70	30
40	50	50
65	50	50
70	2	98
75	99	1
80	99	1

3.3 数据分析

高分辨液质采集的数据通过 CD 3.3 (Compound Discoverer 3.3)(Thermo Fisher)完成数据初步整理后进行数据库 mzCloud 和 mzVault 检索比对。

4 结果

样本在数据库中共匹配到 2525 个化合物。

质谱数据库如下图示，结果详见附件 一：

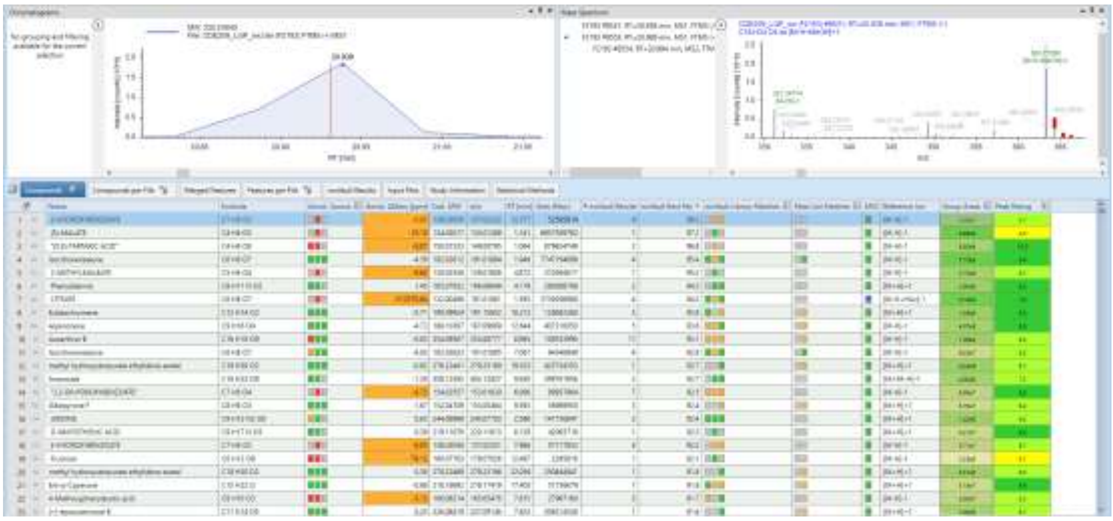

对 mzCloudt 中匹配到的所有物质提供一级二级谱图，详见附件二。

5 附件与帮助信息

5.1 项目结果文件

（1）附件 1：化合物鉴定结果列表 (含总表和三级表)

（2）附件 2： 鉴定的化合物结构谱图信息

5.2 参考文献

1 QE 仪 器 性 能 信 息 ：
https://www.thermofisher.com/order/catalog/product/IQLAAEGAAPFALGMAZR

2 CD 数据库检索设置

3 KEGG 公共访问链接： https://www.kegg.jp/

4 附件辅助信息

#	表头参数	含义
1	Name	数据库中鉴定到的化合物名称
2	Formula	数据库中鉴定到的化合物化学分子式
3	Annot. ΔMass [Da]	分子量差
4	Calc. MW	校正后的分子量
5	Annotation MW	理论分子量
6	m/z	质荷比

7	RT [min]	色谱保留时间
8	mzVault Results	mzVault 数据库匹配丰度
9	mzVault Best Match	mzVault 数据库匹配度打分（值越高鉴定到的结果可信度越高）
10	Reference Ion	正/负加和离子
11	Area	组分峰面积
