# Supplementary material for: Combined treatment with Acorus tatarinowii Schott and Panax notoginseng saponins ameliorates brain–gut axis dysfunction in MCAO/R rats with suppression of TLR4/MyD88/NF-κB signaling and associated gut microbiota changes
Source: Front Pharmacol. 2026 Jun 29;17:1683558. doi: 10.3389/fphar.2026.1683558 (PMC13357153; doi:10.3389/fphar.2026.1683558)
Supplement: Supplementary file 2 [file DataSheet1.zip › Supplementary_Materials/Supplementary_Data_S1C_Q-Orbitrap_Total_Ion_Chromatogram.docx]

**CDF014样本中天然产物鉴定总离子流图谱**

注：第1栏为正离子模式总离子流图，第2栏为负离子模式总离子流图
