# Supplementary material for: Combined treatment with Acorus tatarinowii Schott and Panax notoginseng saponins ameliorates brain–gut axis dysfunction in MCAO/R rats with suppression of TLR4/MyD88/NF-κB signaling and associated gut microbiota changes
Source: Front Pharmacol. 2026 Jun 29;17:1683558. doi: 10.3389/fphar.2026.1683558 (PMC13357153; doi:10.3389/fphar.2026.1683558)
Supplement: Supplementary file 2 [file DataSheet1.zip › Supplementary_Materials/Supplementary_Data_S1D_HPLC_Quantification_Report.pdf]

中药提取液中α-细辛脑、β-细辛醚的含量检测报告

研究单位：武汉赛维尔生物科技有限公司

研究单位地址：武汉东湖高新区高新二路388号生物医药园22栋5楼

委托单位及委托人：郝琳瑶

项目研究人：胡飞

联系方式：15207118928

项目开始日期：2026.01.28

项目结束日期：2026.02.10

目录：

1. 实验目的：......3
2. 实验准备：......3
2.1 实验试剂与耗材：......3
2.2 实验仪器......3
3. 实验方法......4
3.1 液相色谱方法......4
3.2 标准品的配制......4
3.3 样品处理方法......5
3.4 数据计算：......5
4. 实验结果：......5
4.1 标准品结果......5
4.2 样本结果......6

1. 实验目的：

采用高效液相色谱方法进行样本中α-细辛脑、β-细辛醚的定量实验。

样本：1个中药提取液样本。

2. 实验准备：

2.1 实验试剂与耗材：

α-细辛脑、β-细辛醚：对照品，客户提供

EP管：1.5 mL，武汉赛维尔生物科技有限公司

乙腈：色谱级，Thermo

甲醇：色谱级，Thermo

2.2 实验仪器

半微量电子天平：MS105DU，梅特勒·托利多国际贸易有限公司

纯水/超纯水一体机系统：Direct-Q®5，德国默克密理博

高速微量冷冻离心机：D3024R，北京大龙兴创实验仪器有限公司

涡旋振荡器：MX-F，武汉赛维尔生物科技有限公司

移液器：0.5~10 μL，2~20 μL，20~200 μL，100~1000 μL，Eppendorf

色谱仪：Agilent 1260 Infinity II，安捷伦科技有限公司

3. 实验方法

3.1 液相色谱方法

色谱柱 Welch Ultimate PLUS C18 250×4.6 mm, 5 μm；DAD 检测器，检测波长为 257nm；流速 1 mL/min；柱温 35 ℃，进样量为 5μL。

流动相的配制：流动相 A 为 0.1%三氟乙酸水溶液，流动相 B 为乙腈，采用下列梯度洗脱。

Table 2 流动相梯度

时间（min）	水相比例（%）	有机相比例（%）
10	99	1
20	70	30
30	70	30
40	50	50
65	50	50
70	2	98
75	99	1
80	99	1

3.2 标准品的配制

分别称取一定量的α-细辛脑、β-细辛醚标准品粉末，用甲醇配制成 200ug/mL 的混合溶液，上机检测，记录色谱图。

3.3 样品处理方法

将中药提取液样本涡旋混合均匀，取 200uL 加入 800uL 甲醇提取，加入 3 颗研磨珠，研磨 5 min，涡旋混匀 10 min，13000 rpm 离心 10 min，取上清上机分析，记录色谱图。

3.4 数据计算：

化合物的色谱图采集和积分由软件 Chemstation 进行处理。

4. 实验结果：

4.1 标准品结果

分析物名称	含量 [ug/mL]	峰面积 [mAU*s]
α细辛脑	200	620.2242
β细辛醚	200	825.3713

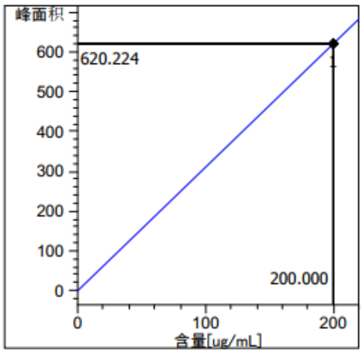

α细辛脑 预期 RT: 44.738
DAD1 A, Sig=257,4 Ref=off
相关性: 1.00000
残留标准误差: 0.00000
公式: y = mx + b
m: 3.10112
b: 0.00000
x: 含量[ug/mL]
y: 峰面积

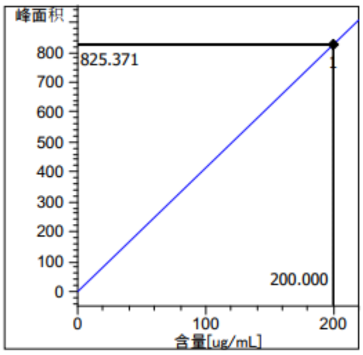

β细辛醚 预期 RT: 46.200
DAD1 A, Sig=257,4 Ref=off
相关性: 1.00000
残留标准误差: 0.00000
公式: y = mx + b
m: 4.12686
b: 0.00000
x: 含量[ug/mL]
y: 峰面积

4.2 样本结果

样本中芦丁的检测结果汇总

样本编号	检测浓度 [ug/mL]		稀释倍数	实际浓度 [ug/mL]	
	α细辛脑	β细辛醚		α细辛脑	β细辛醚
样本 1	368.5792	7.87637	5	1842.90	39.38
样本 2	368.2161	7.85054	5	1841.08	39.25
样本 3	369.1578	7.8491	5	1845.79	39.25

详见附件。
