# Supplementary material for: Combined treatment with Acorus tatarinowii Schott and Panax notoginseng saponins ameliorates brain–gut axis dysfunction in MCAO/R rats with suppression of TLR4/MyD88/NF-κB signaling and associated gut microbiota changes
Source: Front Pharmacol. 2026 Jun 29;17:1683558. doi: 10.3389/fphar.2026.1683558 (PMC13357153; doi:10.3389/fphar.2026.1683558)
Supplement: Supplementary file 2 [file DataSheet1.zip › Supplementary_Materials/Supplementary_File_S1_Botanical Drug Characterization.docx]

**Supplementary File S1**

**Botanical Drug Characterization and Orthogonal Fingerprinting of *Acorus tatarinowii* Schott Extract**

**1. Scope and source files**

This supplementary file summarizes the orthogonal analytical characterization of the *Acorus tatarinowii* Schott (AT) extract used in the study. The characterization combines targeted HPLC-DAD quantification of marker constituents with untargeted high-resolution Q-Orbitrap LC-MS/MS chemical profiling. The original analytical reports and raw summary files are provided as Supplementary Data Files S1A-S1E in the accompanying supplementary package.

| **Supplementary item** | **Content** | **Analytical platform** | **Purpose** |
| --- | --- | --- | --- |
| Supplementary File S1 | Curated botanical drug characterization summary | Compiled report | Submission-ready summary of orthogonal fingerprinting |
| Supplementary Data S1A | Original Q-Orbitrap HR-LC-MS/MS report | Thermo UltiMate 3000 RS + Q Exactive | Untargeted chemical profiling of AT extract |
| Supplementary Data S1B | Q-Orbitrap compound identification list | Compound Discoverer 3.3 database matching | Complete list of database-matched compounds |
| Supplementary Data S1C | Total ion chromatogram document | Q-Orbitrap HR-LC-MS/MS | Representative positive- and negative-ion TIC profiles |
| Supplementary Data S1D | Original HPLC-DAD report | Agilent 1260 Infinity II DAD | Targeted quantification of α-asarone and β-asarone |
| Supplementary Data S1E | HPLC quantification data summary | ChemStation integration output | Replicate concentrations and calculated marker contents |

**2. Targeted HPLC-DAD quantification of marker constituents**

Targeted HPLC-DAD analysis was performed to quantify α-asarone and β-asarone in the AT extract. The assay used reference standards for both analytes, and the samples were processed in triplicate. The quantitative results are summarized below. Values are reported in µg/mL for the extract solution analyzed in the original report.

Supplementary Table SF1-1. HPLC-DAD conditions for targeted quantification of α-asarone and β-asarone.

| **Parameter** | **Condition** |
| --- | --- |
| Instrument | Agilent 1260 Infinity II with DAD detector |
| Column | Welch Ultimate PLUS C18, 250 × 4.6 mm, 5 µm |
| Detection wavelength | 257 nm |
| Flow rate | 1.0 mL/min |
| Column temperature | 35 °C |
| Injection volume | 5 µL |
| Mobile phase A | 0.1% trifluoroacetic acid in water |
| Mobile phase B | Acetonitrile |
| Sample preparation | 200 µL extract + 800 µL methanol; grinding 5 min, vortex 10 min, centrifugation at 13,000 rpm for 10 min; supernatant injected |

Supplementary Table SF1-2. HPLC-DAD quantification of α-asarone and β-asarone in the AT extract.

| **Sample** | **Measured α-asarone (µg/mL)** | **Measured β-asarone (µg/mL)** | **Dilution factor** | **Actual α-asarone (µg/mL)** | **Actual β-asarone (µg/mL)** |
| --- | --- | --- | --- | --- | --- |
| Replicate 1 | 368.57924 | 7.87637 | 5 | 1842.90 | 39.38 |
| Replicate 2 | 368.21607 | 7.85054 | 5 | 1841.08 | 39.25 |
| Replicate 3 | 369.15784 | 7.84910 | 5 | 1845.79 | 39.25 |
| Mean ± SD | - | - | - | 1843.26 ± 2.37 | 39.29 ± 0.08 |
| RSD (%) | - | - | - | 0.13 | 0.20 |


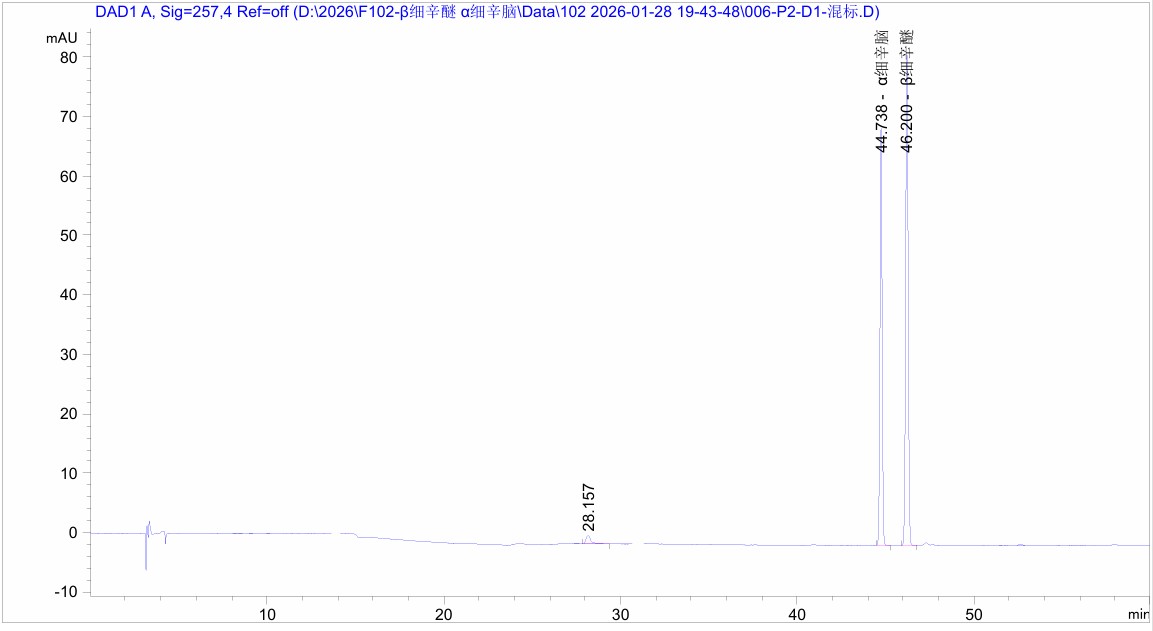


Supplementary Figure SF1-1. Representative HPLC-DAD chromatogram of the mixed α-asarone/β-asarone reference standard.


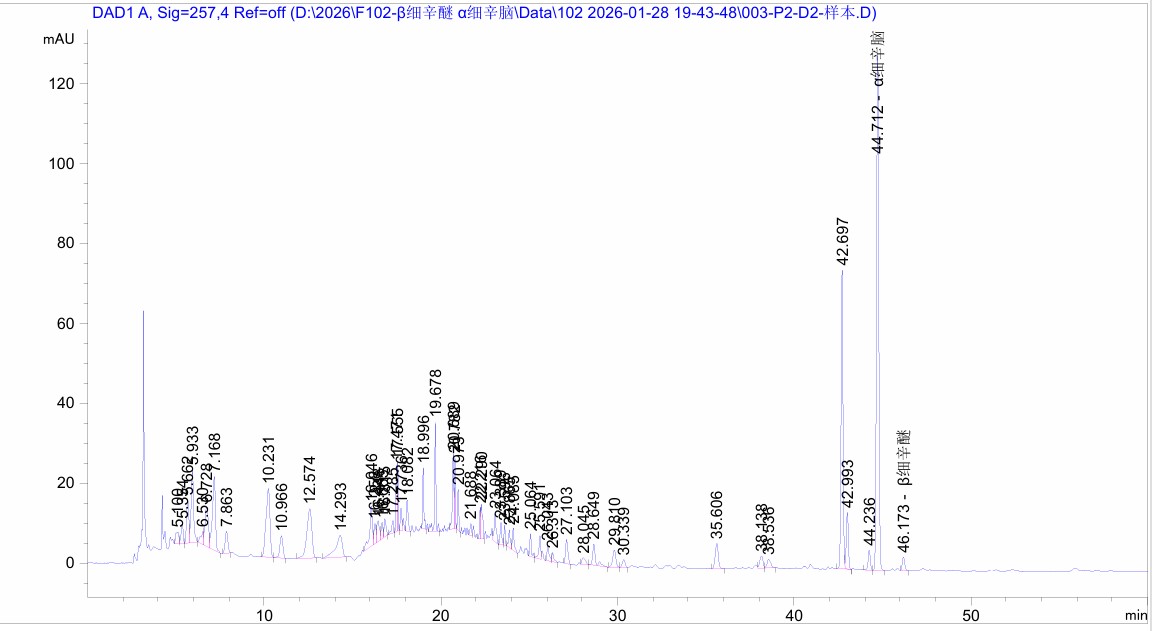


Supplementary Figure SF1-2. Representative HPLC-DAD chromatogram of the AT extract sample.

**3. Q-Orbitrap high-resolution LC-MS/MS chemical profiling**

Untargeted high-resolution LC-MS/MS profiling was performed using a Q Exactive mass spectrometer coupled to an UltiMate 3000 RS LC system. Database matching was performed using Compound Discoverer 3.3 with mzCloud and mzVault library searches. A total of 2525 database-matched compounds were reported in the original compound identification list; 148 features had an mzVault best match score of 90 or above. The complete compound list is supplied as Supplementary Data S1B.

Supplementary Table SF1-3. Q-Orbitrap HR-LC-MS/MS conditions for untargeted chemical profiling.

| **Parameter** | **Condition** |
| --- | --- |
| LC system | Thermo UltiMate 3000 RS |
| MS system | Thermo Q Exactive high-resolution mass spectrometer |
| Ion source | Electrospray ionization (ESI) |
| Scan mode | Positive/negative ion switching |
| Acquisition mode | Full MS/dd-MS2 |
| Resolution | 70,000 for full MS; 17,500 for dd-MS2 |
| Scan range | m/z 100.0-1500.0 |
| Spray voltage | 3.2 kV in positive and negative modes |
| Capillary temperature | 300 °C |
| Collision energy | (N)CE 30, 40, 60 |
| Sheath/auxiliary gas | Nitrogen, 40 Arb / 15 Arb; auxiliary gas temperature 350 °C |
| LC column | Welch Ultimate Plus-C18, 4.6 × 250 mm, 5 µm |
| Flow rate | 0.80 mL/min |
| Mobile phase | 0.1% formic acid in water (A) and acetonitrile (B) |
| Column temperature | 35 °C |
| Injection volume | 5 µL |
| Acquisition time | 80 min |

Supplementary Figure SF1-3. Total ion chromatograms of the AT extract acquired in positive- and negative-ion modes.

Supplementary Table SF1-4. Representative high-confidence compounds from the Q-Orbitrap HR-LC-MS/MS identification list, sorted by mzVault best match score. The complete list is provided as Supplementary Data S1B.

| **No.** | **Name** | **Formula** | **m/z** | **RT (min)** | **mzVault best match** | **Peak area** |
| --- | --- | --- | --- | --- | --- | --- |
| 1 | Choline | C5 H13 N O | 104.10748 | 2.528 | 100.0 | 2.393e+09 |
| 2 | L-Pyroglutamic acid | C5 H7 N O3 | 130.04996 | 6.106 | 100.0 | 1.243e+10 |
| 3 | L-Norleucine | C6 H13 N O2 | 132.10210 | 6.526 | 100.0 | 5.239e+09 |
| 4 | Valine | C5 H11 N O2 | 118.08659 | 3.258 | 100.0 | 2.529e+09 |
| 5 | 4-Oxoproline | C5 H7 N O3 | 128.03404 | 6.085 | 99.8 | 6.932e+09 |
| 6 | 5-Hydroxynicotinic acid | C6 H5 N O3 | 138.01857 | 5.751 | 99.6 | 1.274e+08 |
| 7 | 3-Hydroxypicolinic acid | C6 H5 N O3 | 140.03433 | 5.753 | 99.5 | 8.024e+08 |
| 8 | Betaine | C5 H11 N O2 | 140.06813 | 73.727 | 99.5 | 1.208e+08 |
| 9 | Adenosine | C10 H13 N5 O4 | 268.10367 | 12.260 | 99.4 | 9.465e+09 |
| 10 | L-Aspartic acid | C4 H7 N O4 | 132.02895 | 2.696 | 99.4 | 2.309e+09 |
| 11 | L-(-)-Malic acid | C4 H6 O5 | 133.01300 | 3.766 | 99.1 | 2.253e+10 |
| 12 | DL-Stachydrine | C7 H13 N O2 | 144.10196 | 3.142 | 99.1 | 1.978e+09 |
| 13 | Leucylproline | C11 H20 N2 O3 | 229.15479 | 17.076 | 99.0 | 3.108e+08 |
| 14 | Betaine | C5 H11 N O2 | 118.08648 | 2.860 | 98.8 | 6.667e+09 |
| 15 | L-Threonine | C4 H9 N O3 | 120.06582 | 2.703 | 98.7 | 1.404e+09 |

Supplementary Table SF1-5. Asarone-related database matches detected in the Q-Orbitrap HR-LC-MS/MS profiling dataset. These qualitative matches complement the targeted HPLC-DAD quantification.

| **Name** | **Formula** | **m/z** | **RT (min)** | **mzVault best match** | **Peak area** |
| --- | --- | --- | --- | --- | --- |
| β-Asarone | C12 H16 O3 | 209.11723 | 44.914 | 91.4 | 4.126e+08 |
| β-Asarone | C12 H16 O3 | 209.11728 | 28.547 | 88.8 | 4.763e+08 |
| β-Asarone | C12 H16 O3 | 209.11729 | 43.131 | 88.7 | 5.798e+07 |
| β-Asarone | C12 H16 O3 | 209.11729 | 70.239 | 88.6 | 1.113e+08 |
| β-Asarone | C12 H16 O3 | 209.11742 | 42.906 | 88.4 | 2.560e+08 |
| β-Asarone | C12 H16 O3 | 209.11726 | 37.753 | 86.5 | 1.004e+08 |
| β-Asarone β-细辛醚 | C12 H16 O3 | 209.11737 | 69.553 | 83.8 | 2.750e+07 |
| β-Asarone | C12 H16 O3 | 209.11734 | 21.604 | 83.5 | 1.827e+07 |

**4. Interpretation and submission note**

The HPLC-DAD assay provides targeted quantitative confirmation of marker constituents in the AT extract, while the Q-Orbitrap HR-LC-MS/MS analysis provides an orthogonal high-resolution chemical profile and total ion chromatographic fingerprint. These data support batch-level characterization of the AT extract used in the study. The Q-Orbitrap identifications are database matches and should be interpreted as qualitative annotations unless independently confirmed by authentic reference standards. The targeted HPLC-DAD assay provides the quantitative marker content data for α-asarone and β-asarone.
