# Supplementary material for: Combined treatment with Acorus tatarinowii Schott and Panax notoginseng saponins ameliorates brain–gut axis dysfunction in MCAO/R rats with suppression of TLR4/MyD88/NF-κB signaling and associated gut microbiota changes
Source: Front Pharmacol. 2026 Jun 29;17:1683558. doi: 10.3389/fphar.2026.1683558 (PMC13357153; doi:10.3389/fphar.2026.1683558)
Supplement: Supplementary file 2 [file DataSheet1.zip › Supplementary_Materials/Supplementary_File_S1_Botanical Drug Characterization.pdf]

Supplementary File S1

Botanical Drug Characterization and Orthogonal Fingerprinting of *Acorus tatarinowii* Schott Extract

1. Scope and source files

This supplementary file summarizes the orthogonal analytical characterization of the *Acorus tatarinowii* Schott (AT) extract used in the study. The characterization combines targeted HPLC-DAD quantification of marker constituents with untargeted high-resolution Q-Orbitrap LC-MS/MS chemical profiling. The original analytical reports and raw summary files are provided as Supplementary Data Files S1A-S1E in the accompanying supplementary package.

Supplementary item	Content	Analytical platform	Purpose
Supplementary File S1	Curated botanical drug characterization summary	Compiled report	Submission-ready summary of orthogonal fingerprinting
Supplementary Data S1A	Original Q-Orbitrap HR-LC-MS/MS report	Thermo UltiMate 3000 RS + Q Exactive	Untargeted chemical profiling of AT extract
Supplementary Data S1B	Q-Orbitrap compound identification list	Compound Discoverer 3.3 database matching	Complete list of database-matched compounds
Supplementary Data S1C	Total ion chromatogram document	Q-Orbitrap HR-LC-MS/MS	Representative positive- and negative-ion TIC profiles
Supplementary Data S1D	Original HPLC-DAD report	Agilent 1260 Infinity II DAD	Targeted quantification of α-asarone and β-asarone
Supplementary Data S1E	HPLC quantification data summary	ChemStation integration output	Replicate concentrations and calculated marker contents

2. Targeted HPLC-DAD quantification of marker constituents

Targeted HPLC-DAD analysis was performed to quantify α-asarone and β-asarone in the AT extract. The assay used reference standards for both analytes, and the samples were processed in triplicate. The quantitative results are summarized below. Values are reported in µg/mL for the extract solution analyzed in the original report.

Supplementary Table SF1-1. HPLC-DAD conditions for targeted quantification of α-asarone and β-asarone.

Parameter	Condition
Instrument	Agilent 1260 Infinity II with DAD detector

Parameter	Condition
Column	Welch Ultimate PLUS C18, 250 × 4.6 mm, 5 µm
Detection wavelength	257 nm
Flow rate	1.0 mL/min
Column temperature	35 °C
Injection volume	5 µL
Mobile phase A	0.1% trifluoroacetic acid in water
Mobile phase B	Acetonitrile
Sample preparation	200 µL extract + 800 µL methanol; grinding 5 min, vortex 10 min, centrifugation at 13,000 rpm for 10 min; supernatant injected

Supplementary Table SF1-2. HPLC-DAD quantification of α-asarone and β-asarone in the AT extract.

Sample	Measured α-asarone (µg/mL)	Measured β-asarone (µg/mL)	Dilution factor	Actual α-asarone (µg/mL)	Actual β-asarone (µg/mL)
Replicate 1	368.57924	7.87637	5	1842.90	39.38
Replicate 2	368.21607	7.85054	5	1841.08	39.25
Replicate 3	369.15784	7.84910	5	1845.79	39.25
Mean ± SD	-	-	-	1843.26 ± 2.37	39.29 ± 0.08
RSD (%)	-	-	-	0.13	0.20

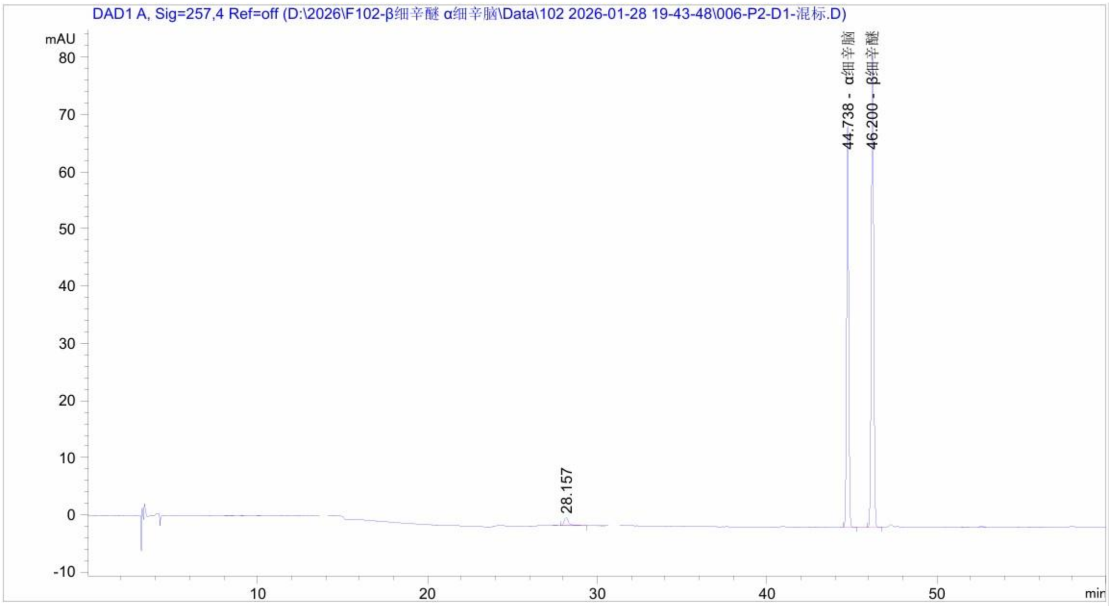

Supplementary Figure SF1-1. Representative HPLC-DAD chromatogram of the mixed α-asarone/β-asarone reference standard.

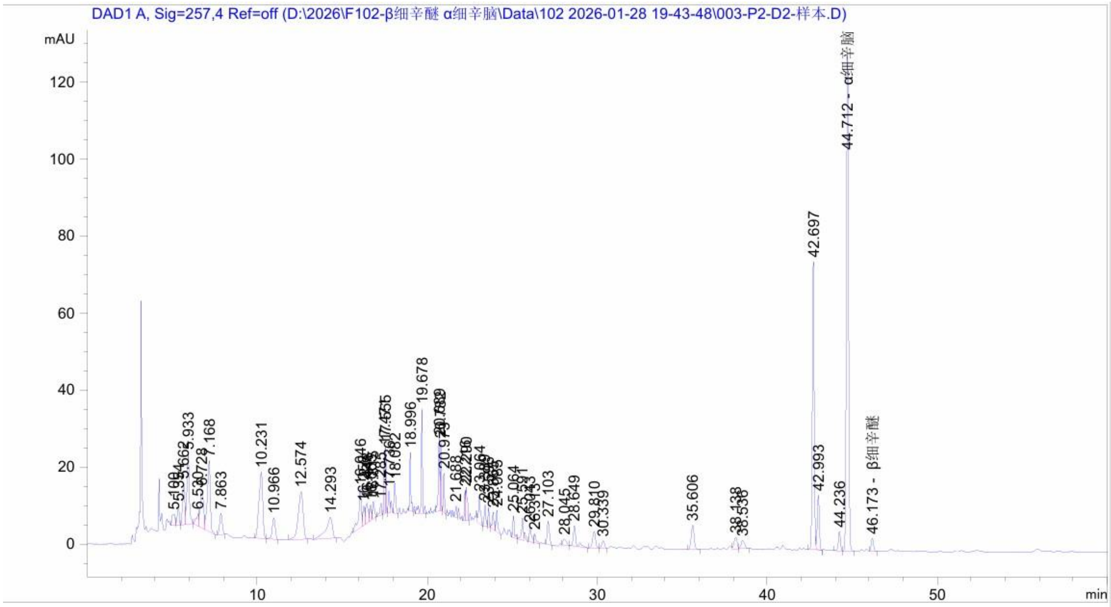

Supplementary Figure SF1-2. Representative HPLC-DAD chromatogram of the AT extract sample.

3. Q-Orbitrap high-resolution LC-MS/MS chemical profiling

Untargeted high-resolution LC-MS/MS profiling was performed using a Q Exactive mass spectrometer coupled to an UltiMate 3000 RS LC system. Database matching was performed using Compound Discoverer 3.3 with mzCloud and mzVault library searches. A total of 2525 database-matched compounds were reported in the original compound identification list; 148 features had an mzVault best match score of 90 or above. The complete compound list is supplied as Supplementary Data S1B.

Supplementary Table SF1-3. Q-Orbitrap HR-LC-MS/MS conditions for untargeted chemical profiling.

Parameter	Condition
LC system	Thermo UltiMate 3000 RS
MS system	Thermo Q Exactive high-resolution mass spectrometer
Ion source	Electrospray ionization (ESI)
Scan mode	Positive/negative ion switching
Acquisition mode	Full MS/dd-MS2
Resolution	70,000 for full MS; 17,500 for dd-MS2
Scan range	m/z 100.0-1500.0
Spray voltage	3.2 kV in positive and negative modes
Capillary temperature	300 °C
Collision energy	(N)CE 30, 40, 60
Sheath/auxiliary gas	Nitrogen, 40 Arb / 15 Arb; auxiliary gas temperature 350 °C
LC column	Welch Ultimate Plus-C18, 4.6 × 250 mm, 5 µm
Flow rate	0.80 mL/min
Mobile phase	0.1% formic acid in water (A) and acetonitrile (B)
Column temperature	35 °C
Injection volume	5 µL
Acquisition time	80 min

Supplementary Figure SF1-3. Total ion chromatograms of the AT extract acquired in positive- and negative-ion modes.

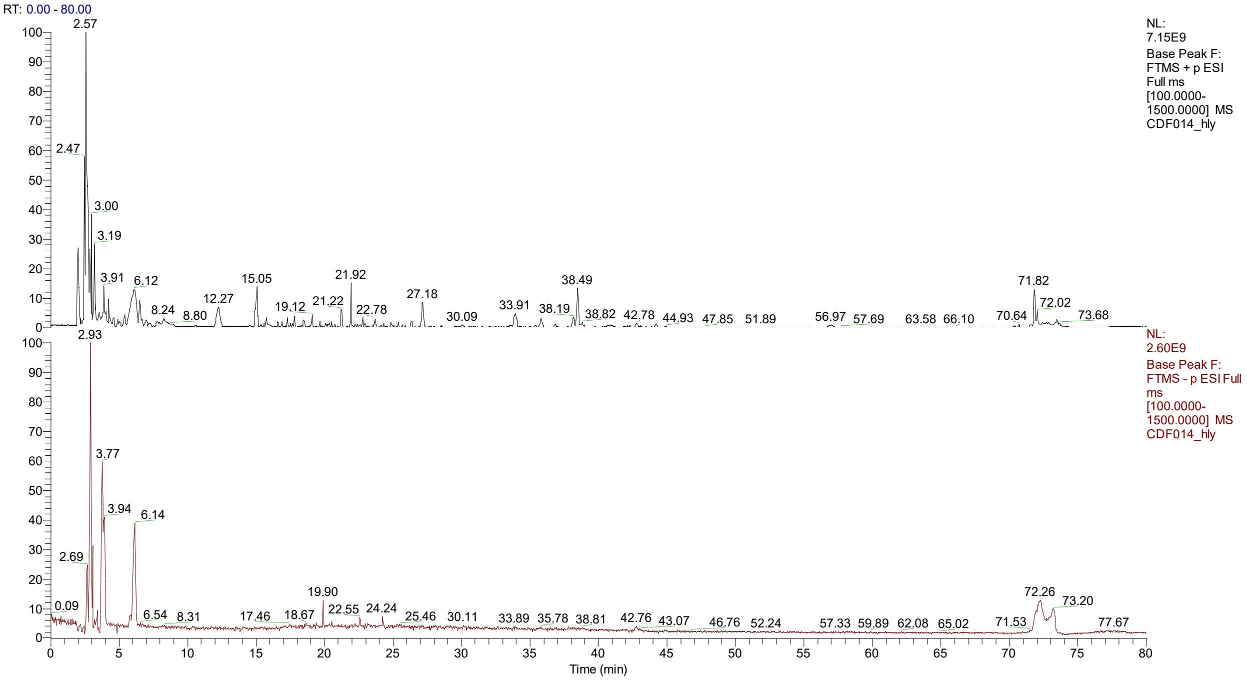

Supplementary Table SF1-4. Representative high-confidence compounds from the Q-Orbitrap HR-LC-MS/MS identification list, sorted by mzVault best match score. The complete list is provided as Supplementary Data S1B.

No.	Name	Formula	m/z	RT (min)	mzVault best match	Peak area
1	Choline	C5 H13 N O	104.10748	2.528	100.0	2.393e+09
2	L-Pyroglutamic acid	C5 H7 N O3	130.04996	6.106	100.0	1.243e+10
3	L-Norleucine	C6 H13 N O2	132.10210	6.526	100.0	5.239e+09
4	Valine	C5 H11 N O2	118.08659	3.258	100.0	2.529e+09
5	4-Oxoproline	C5 H7 N O3	128.03404	6.085	99.8	6.932e+09
6	5-Hydroxynicotinic acid	C6 H5 N O3	138.01857	5.751	99.6	1.274e+08
7	3-Hydroxypicolinic acid	C6 H5 N O3	140.03433	5.753	99.5	8.024e+08
8	Betaine	C5 H11 N O2	140.06813	73.727	99.5	1.208e+08
9	Adenosine	C10 H13 N5 O4	268.10367	12.260	99.4	9.465e+09
10	L-Aspartic acid	C4 H7 N O4	132.02895	2.696	99.4	2.309e+09
11	L-(-)-Malic acid	C4 H6 O5	133.01300	3.766	99.1	2.253e+10

No.	Name	Formula	m/z	RT (min)	mzVault best match	Peak area
12	DL-Stachydrine	C7 H13 N O2	144.10196	3.142	99.1	1.978e+09
13	Leucylproline	C11 H20 N2 O3	229.15479	17.076	99.0	3.108e+08
14	Betaine	C5 H11 N O2	118.08648	2.860	98.8	6.667e+09
15	L-Threonine	C4 H9 N O3	120.06582	2.703	98.7	1.404e+09

Supplementary Table SF1-5. Asarone-related database matches detected in the Q-Orbitrap HR-LC-MS/MS profiling dataset. These qualitative matches complement the targeted HPLC-DAD quantification.

Name	Formula	m/z	RT (min)	mzVault best match	Peak area
β-Asarone	C12 H16 O3	209.11723	44.914	91.4	4.126e+08
β-Asarone	C12 H16 O3	209.11728	28.547	88.8	4.763e+08
β-Asarone	C12 H16 O3	209.11729	43.131	88.7	5.798e+07
β-Asarone	C12 H16 O3	209.11729	70.239	88.6	1.113e+08
β-Asarone	C12 H16 O3	209.11742	42.906	88.4	2.560e+08
β-Asarone	C12 H16 O3	209.11726	37.753	86.5	1.004e+08
β-Asarone β-细辛醚	C12 H16 O3	209.11737	69.553	83.8	2.750e+07
β-Asarone	C12 H16 O3	209.11734	21.604	83.5	1.827e+07

4. Interpretation and submission note

The HPLC-DAD assay provides targeted quantitative confirmation of marker constituents in the AT extract, while the Q-Orbitrap HR-LC-MS/MS analysis provides an orthogonal high-resolution chemical profile and total ion chromatographic fingerprint. These data support batch-level characterization of the AT extract used in the study. The Q-Orbitrap identifications are database matches and should be interpreted as qualitative annotations unless independently confirmed by authentic reference standards. The targeted HPLC-DAD assay provides the quantitative marker content data for α-asarone and β-asarone.
