# Supplementary figures and images for: Combined treatment with Acorus tatarinowii Schott and Panax notoginseng saponins ameliorates brain–gut axis dysfunction in MCAO/R rats with suppression of TLR4/MyD88/NF-κB signaling and associated gut microbiota changes
Source: Front Pharmacol. 2026 Jun 29;17:1683558. doi: 10.3389/fphar.2026.1683558 (PMC13357153; doi:10.3389/fphar.2026.1683558)

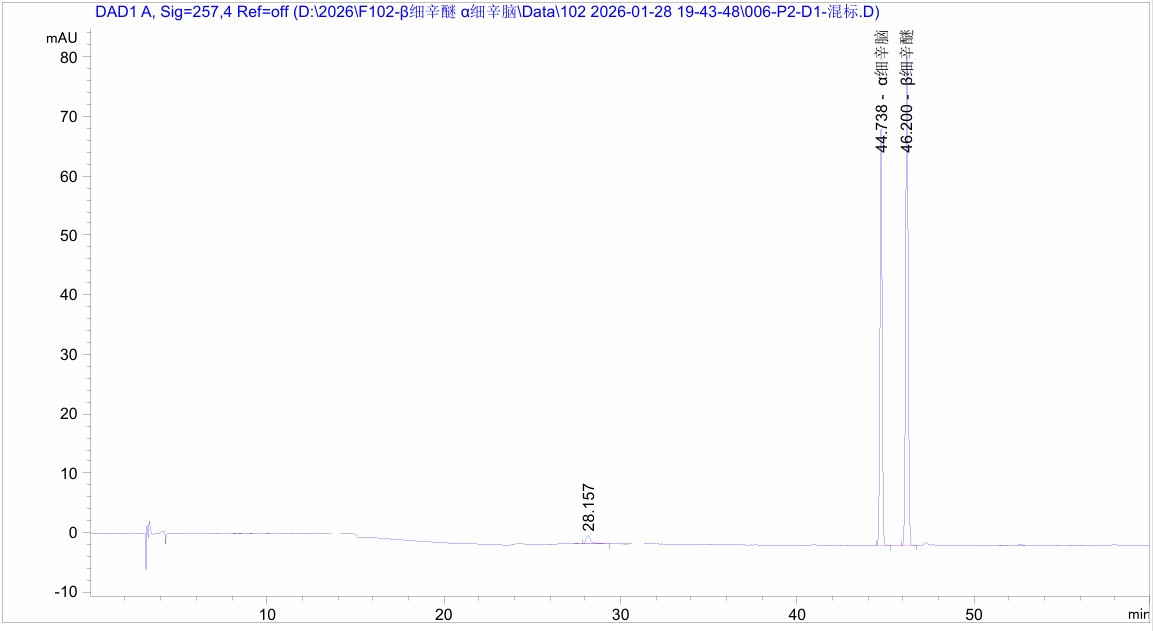

Supplement: Supplementary file 2 [file DataSheet1.zip › Supplementary_Materials/Figure_SF1-1_HPLC_mixed_standard.png.jpg]

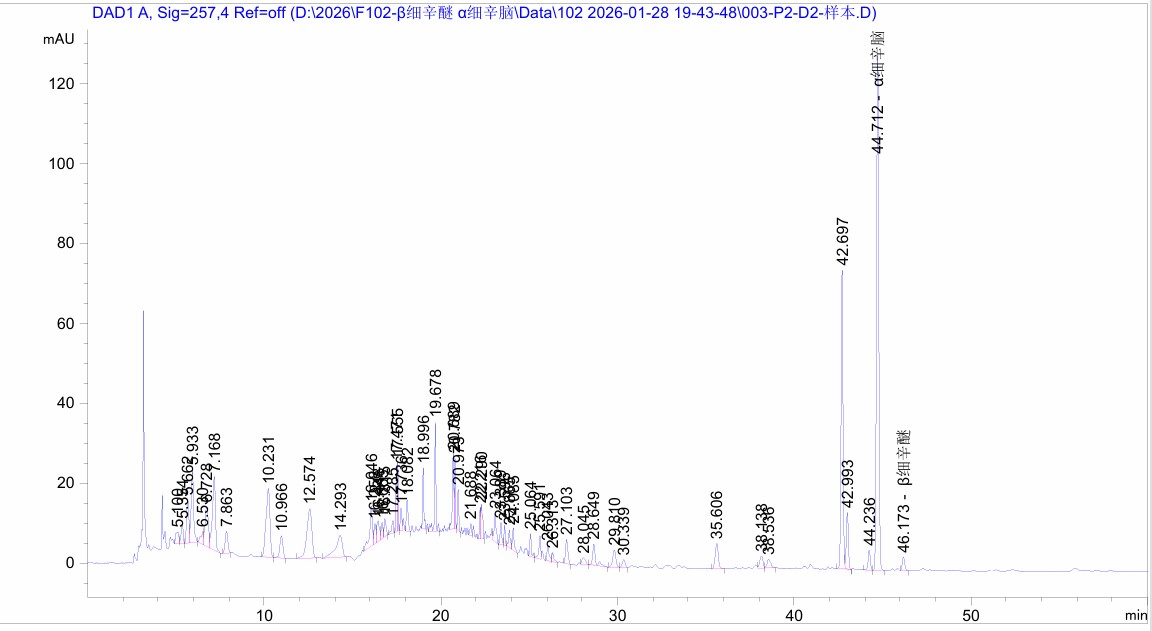

Supplement: Supplementary file 2 [file DataSheet1.zip › Supplementary_Materials/Figure_SF1-2_HPLC_sample.jpg]

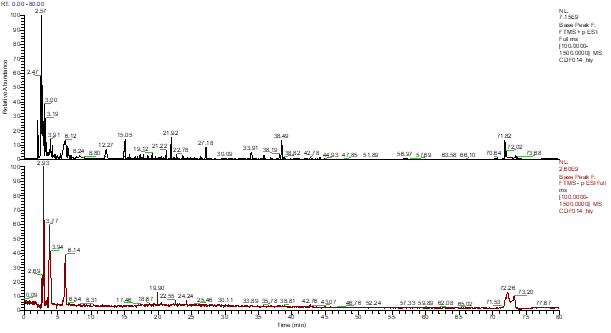

Supplement: Supplementary file 2 [file DataSheet1.zip › Supplementary_Materials/Figure_SF1-3_QOrbitrap_TIC.tif]
